# Identifying Signal-Crosstalk Mechanism in Maize Plants during Combined Salinity and Boron Stress Using Integrative Systems Biology Approaches

**DOI:** 10.1155/2022/1027288

**Published:** 2022-04-18

**Authors:** Drishtee Barua, Asutosh Mishra, P. B. Kirti, Pankaj Barah

**Affiliations:** ^1^Department of Molecular Biology and Biotechnology, Tezpur University, Assam, 784028, India; ^2^Agri Biotech Foundation, Agricultural University Campus, Rajendranagar, Hyderabad, 500030, India

## Abstract

Combined stress has been seen as a major threat to world agriculture production. Maize is one of the leading cereal crops of the world due to its wide spectrum of growth conditions and is moderately sensitive to salt stress. A saline soil environment is a major factor that hinders its growth and overall yield and causes an increase in the concentration of micronutrients like boron, leading to excess over the requirement of the plant. Boron toxicity combined with salinity has been reported to be a serious threat to the yield and quality of maize. The response signatures of the maize plants to the combined effect of salinity and boron stress have not been studied well. We carried out an integrative systems-level analysis of the publicly available transcriptomic data generated on tolerant maize (Lluteño maize from the Atacama Desert, Chile) landrace under combined salt and boron stress. We identified significant biological processes that are differentially regulated in combined salt and boron stress in the leaves and roots of maize, respectively. Protein-protein interaction network analysis identified important roles of *aldehyde dehydrogenase* (ALDH), *galactinol synthase 2* (GOLS2) *proteins of leaf and proteolipid membrane potential regulator* (pmpm4), *metallothionein lea protein group 3* (mlg3), and *cold regulated 410* (COR410) proteins of root in salt tolerance and regulating boron toxicity in maize. Identification of transcription factors coupled with regulatory network analysis using machine learning approach identified a few heat shock factors (HSFs) and *NAC* (*NAM* (no apical meristem, Petunia), *ATAF1–2* (*Arabidopsis thaliana* activating factor), and *CUC2* (cup-shaped cotyledon, *Arabidopsis*)) family transcription factors (TFs) to play crucial roles in salt tolerance, maintaining reactive oxygen species (ROS) levels and minimizing oxidative damage to the cells. These findings will provide new ways to design targeted functional validation experiments for developing multistress-resistant maize crops.

## 1. Introduction

The recent trends in the global population have shown an increase of up to 150 million while the overall gross domestic product (GDP) has reduced by 5% [1]. India is ranked 101^st^ among the 126 countries with a global hunger index (GHI) score of 27.5 which falls under the “serious” category, making it a topic of immediate concern [2]. Global food security has become a serious concern, with the periodic climate change which comes with direct and indirect adverse effects through temperature rise, rainfall, reduction of groundwater levels, soil erosion, flooding, etc. on agricultural production of important crops like rice, wheat, maize, barley, and soybean [3]. This change in climate also helps to increase the incidence of diseases and pests making the agricultural plants vulnerable to them [4]. Being sessile and grown under a direct environment, plants are constantly exposed to different types of severe environmental stresses, i.e., abiotic that are governed by non-living factors like soil salinity, temperature, water, and biotic, governed by living pathogens [5].

Salinity stress is one of the most common stress conditions which affects about 20-50% of irrigated lands worldwide and causes an annual economic loss of up to 12.6 billion dollars [6]. Salinity stress is generally governed by water-soluble salts present in the soil that affect the plant by causing reduction of water and osmotic potential, chlorophyll, etc. leading to death [7]. Reports say that more than 800 million hectares of land have been affected by salinity, and some of them have been rendered completely unusable for agriculture [8]. Roots being the only route for mineral transport to the plants, ions generated from salt enter the plant body via transporters, with their excess transport leading to internal damages to the plant [9]. Boron is an important micronutrient for plants. Deficiency as well as excess amounts of boron acts as two different stress conditions that hamper plant growth and development and restrict crop production worldwide [10]. Different regions around the globe like California, India, Chile, Peru, Malaysia, the Middle East, and some parts of Australia contain an adequately high amount of boron in the soil restricting agricultural production in these areas. Plants, in fact, experience combined stress in the natural environment and respond accordingly, which is quite different from the response to single stress conditions. In the natural environment, soil salinity is considered to be a companion of boron, as it is accumulated as sodium salts in high saline areas due to its soluble nature. This combination of boron and salinity stress is called “BorSal.” BorSal can occur naturally through high boron and saline soil or irrigation with high salt and boron containing water [5, 6, 11, 12].

Maize, one of the most important cereals in the world, not only finds use as a source of food but also as a source of feed for animals and as industrial raw material. It has proved to be the most versatile cereal crop due to its adaptability in various climatic zones and 83% of its production being used in feed, starch, and biofuel industries. Despite its range of adaptability, maize cultivation in the world is limited by diseases and abiotic stress factors leading to a grain loss of about 11% of the total production. As maize is a moderately salt-sensitive crop, salinity stress considerably affects its growth and development and thereby reduces overall yield, which is a matter of immediate concern.

Since the past decade, along with experimental analysis, scientists have been using high-throughput omic approaches to understand the systemic complexity and gain mechanistic insights of plants during stress conditions [13]. The transcriptomic approach for studying the expression and regulation of coding regions of the plant genome in response to different stress conditions has aided in capturing significant information on stress resistance genes and transcription factors [14, 15]. Transcriptomic study of maize during single heat stress has identified 167 putative transcription factors belonging to different TF families like *myeloblastosis* (MYB), *apetala2-ethylene responsive element binding protein* (AP2-EREBP), *basic leucine zipper* (b-ZIP), *basic helix loop helix* (bHLH), *NAC*, and *WRKY* (a TF family renamed with singlet codes of tryptophan, arginine, lysine, and tyrosine) [16]. Maize seeds treated with zinc are reported to improve tolerance against boron toxicity by activating genes related to carbon metabolism, hormone signal transduction, ribosome assembly, etc. [17]. Similarly, the application of salicylic acid improved ROS scavenging systems in the plant during boron stress [18]. Li et al. [19] performed a global transcriptome analysis of maize during individual drought, salinity, heat, and cold stress and reported 167 commonly regulated genes with 10 upregulated and 2 downregulated TFs, respectively. The majority of these analyses reported results targeting a single stress response. A limited number of transcriptomic studies have been conducted in maize against combined stress response, namely, drought and cold stress [20], cold and heat stress [21], and cold and drought stress [22]. The study conducted by Huanca-Mamani et al. [23], on combined salinity and boron stress in maize, focused on their effect in the noncoding regions of maize. Hence, we performed an integrative systems-level transcriptomic data analysis using the publicly available maize dataset to identify the differentially expressed genes along with the common and unique molecular signatures expressed during stress resistance in leaves and roots.

## 2. Materials and Methods

### 2.1. Data Retrieval, Experimental Setup, and Design of Workflow

**Figure 1:**
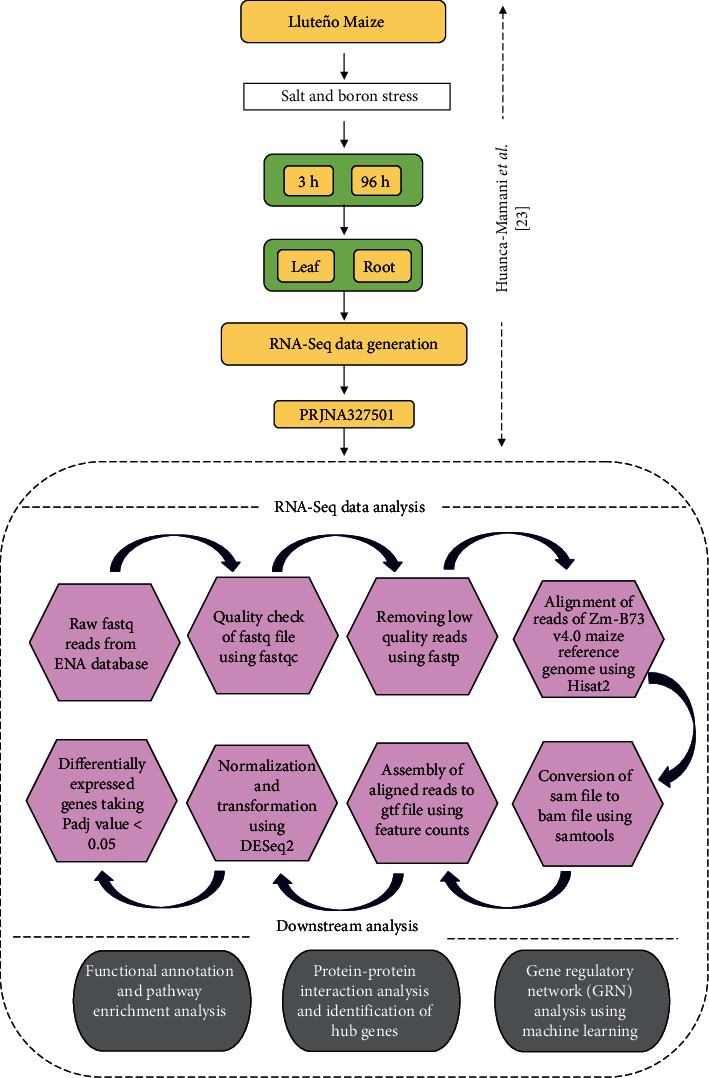
Sequential steps of the RNA-Seq data analysis pipeline, beginning with data retrieval, preprocessing, alignment, assembly, and normalization followed by downstream analysis.

Publicly available RNA-Seq dataset generated on the seeds of Lluteño maize (*Z. mays* L. cv. amylacea) and two commercial hybrids (Prays-214 and GH-2041) by Huanca-Mamani et al. [23] was used for our study. The dataset was retrieved from the NCBI SRA database and downloaded from the ENA server. In the original work [23], seeds of the abovementioned maize variety were first germinated using perlite under greenhouse conditions and allowed to grow for 2 weeks. Two-week-old seedlings were cultured in a hydroponic environment with Hoagland's solution (renewed every 3 days) for 10 days for acclimatization and then subjected to combined NaCl (150 mM) and boron (20 ppm) stress for 3 h and 96 h, respectively. Further, root and leaf samples were collected, stored, and sequenced using the Illumina MiSeq platform generating 150 bp paired-end reads [23]. The data was downloaded in FASTQ format from the ENA BioProject database (Accession number: PRJNA327501) (https://www.ebi.ac.uk/ena/browser/view/PRJNA327501). The dataset contained 6 samples in total, out of which, 4 belonged to combined stress (at 2 time points in root and leaf) and the remaining 2 to the control set. The original study focused on long noncoding RNAs, whereas our analysis is focused on studying the protein-coding genes from the data using our in-house benchmarked RNA-Seq data analysis pipeline (Figure 1).

### 2.2. Transcriptomic Analysis of RNA-Seq Data

**Figure 2:**
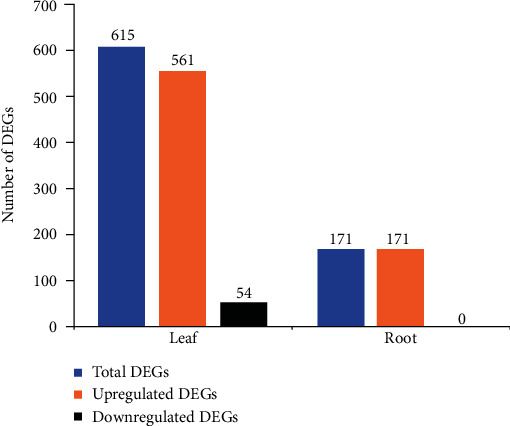
Bar plot showing the number of upregulated, downregulated, and total DEGs in leaf and root, respectively.

**Figure 3:**
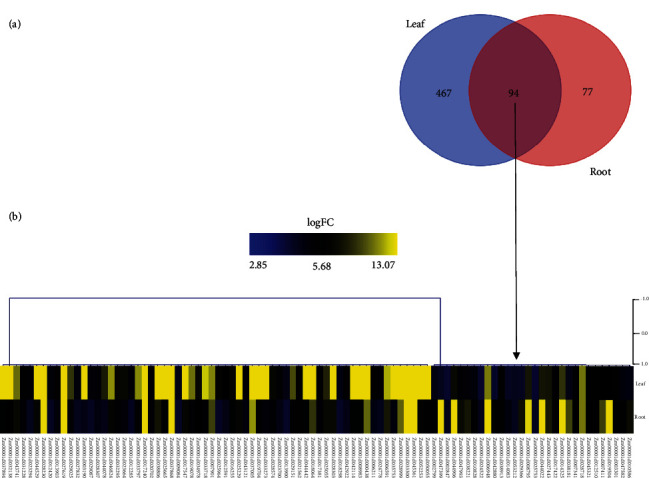
Representation of individual and commonly expressed DEGs in leaf and root. (a) Venn diagram showing overlap of the upregulated DEGs of leaf and root. (b) Heatmap showing expression patterns of the 94 overlapping DEGs in leaf and root of maize. The heat map has been constructed taking the highest and lowest value of expression of a DEG (in terms of log2fold change) in leaf and root, respectively. Blue color represents the lowest, and yellow color represents the highest fold change value.

The transcriptomic data analysis pipeline begins with preprocessing of the data that checks the quality of fastq files done using fastqc [24]. It was followed by fastp to eliminate the presence of any adapters and low-quality reads [25]. The fastq files followed alignment using Hisat2 against the Zm-B73-REFERENCE-GRAMENE-4.0 maize plant reference genome [26] resulting in large sam files. These sam files were then compressed into bam files, and they were subjected to read mapping at the gene level using the featureCounts tool [27] and taking Zea_mays.B73_RefGen_v4.49 as the annotation file (gtf). The count data obtained were subjected to differential expression analysis using the DESeq2 *R* package [28] for the identification of differentially expressed genes (DEGs) (Supplementary Table S1). Taking *p*_adj_ ≤ 0.05, the list of DEGs was created for two datasets (Figure 2). The VennDiagram tool (https://bioinformatics.psb.ugent.be/webtools/Venn/) was used to check for common and unique DEGs in leaf and root (Figure 3(a)). Hierarchical clustering of the overlapping DEGs was done using MeV (version 4.9.0) [29] to observe their expression in leaf and root, respectively (Figure 3(b)). The data was then followed for downstream analysis.

### 2.3. Gene Set Enrichment Analysis and Pathway Analysis

**Figure 4:**
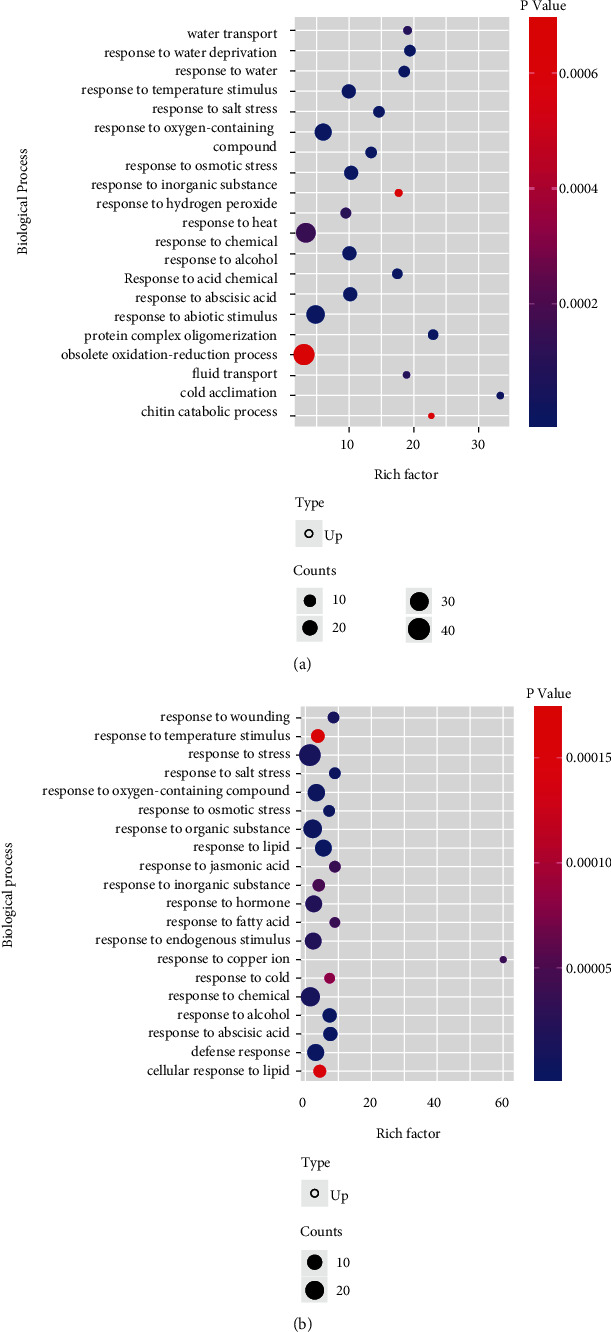
Scatter plot showing gene set enrichment analysis of biological processes in (a) leaf and (b) root.

The DEGs from leaves and roots of maize were subjected to gene ontology analysis for biological processes using g:Profiler, a web server for functional enrichment analysis and conversion of gene list [30]. The gene ontology (GO) term for biological processes was selected after taking *p*_adj_ value <0.05 (Figure 4). Further, pathway enrichment analysis was carried out using the same web server to understand the involvement of DEGs in different biological pathways.

### 2.4. Protein-Protein Interaction (PPI) Networks, Hub Gene Identification, and Mapping of Transcription Factor Families

**Figure 5:**
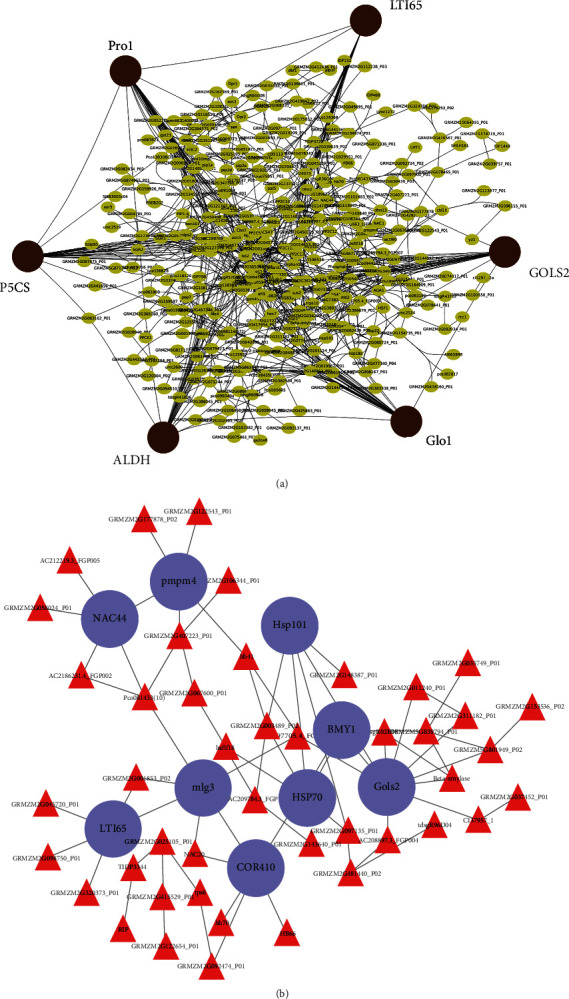
Protein-protein interaction network showing the hub genes with the highest degree centrality in (a) leaf and (b) root.

PPI networks were constructed using the STRING database [31]. Cytoscape (version 3.8.2) was used to visualize the protein interaction relationship network [32]. The network was analyzed using network analyzer, and hub genes were extracted using the degree centrality filter and selecting genes belonging to the top five highest degrees (Figure 5). Functional characterization of these hub genes was done to determine their role in defense response. To determine the presence of transcription factors, the transcripts obtained from PPI analysis were mapped to the list of maize TFs obtained from plant transcription factor database (Plant TFDB) [33] (Table 1).

### 2.5. Using Machine Learning to Construct Transcriptional Regulatory Networks

**Figure 6:**
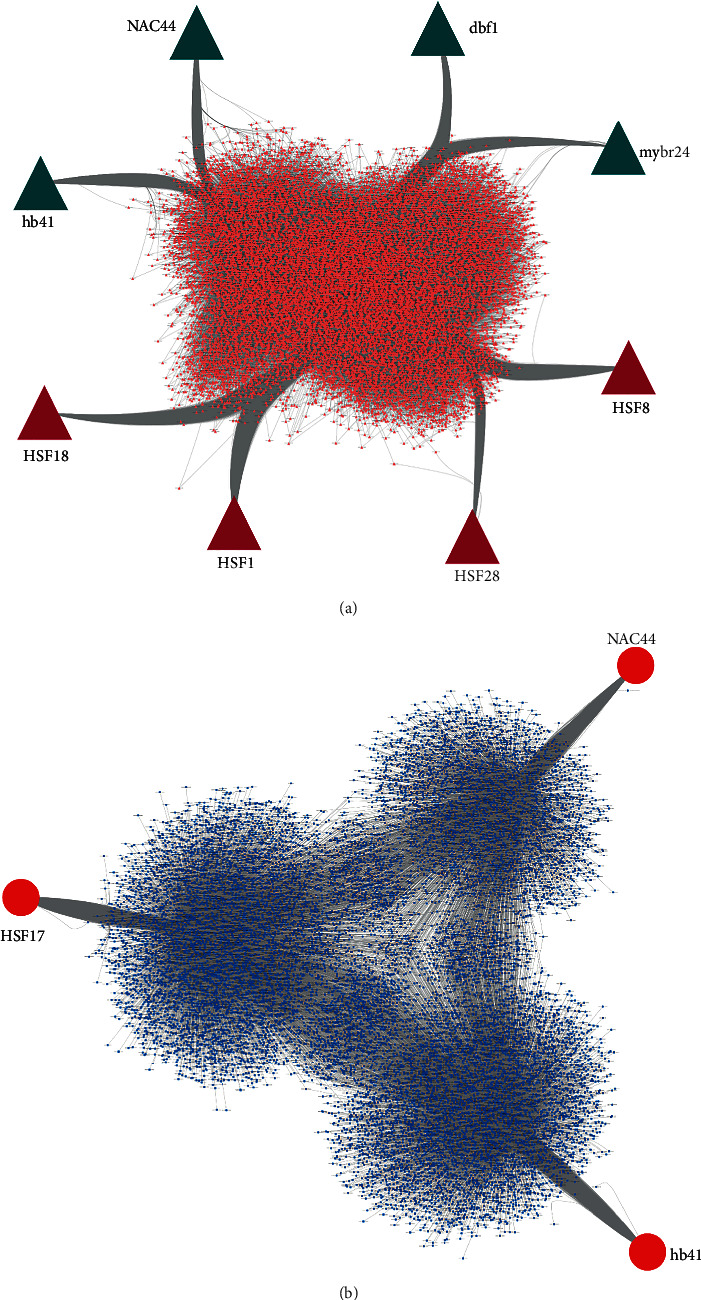
Transcriptional regulatory network showing hub TFs with the highest degree centrality in (a) leaf and (b) root.

Gene regulatory networks for leaf and root of maize were constructed taking significant DEGs as the input. The position weight matrices (PWM) and transcription factor information for maize were downloaded using the *cis*-BP database [34]. The retrieve-seq tool of the RSAT server for Plants was used to retrieve (-1000 bp) upstream regions of the target genes (TG) [35]. The prediction of TFs and their interaction with target genes was carried out using the FIMO tool of the MEME suite, and the results were filtered with a *p* value <10^−4^ [36]. The TF-TG interaction data was represented in the form of an interaction network using Cytoscape [32]. Considering degree centrality as the filtering criteria (>4000), the highly connected transcription factors (TFs) were identified as hubs (Figure 6).

## 3. Results

### 3.1. Identification of DEGs in Maize Leaves and Roots

The analysis of RNA-Seq data of maize revealed a total of 615 protein-coding DEGs in leaf and 171 in root, respectively, among which the number of upregulated DEGs was more than the downregulated DEGs. The overall content of differentially expressed genes revealed a stark difference between leaf and root, with 561 DEGs upregulated in leaf and 171 in the root. On the other hand, 54 DEGs of leaf and no DEGs of root were found to be downregulated (Figure 2). The overexpression of some of these genes might reveal key signatures in defense response against combined stress. The analysis from Venn showed a total of 94 upregulated DEGs commonly expressed in leaf and root (Figure 3(a)). A study of their expression profiles revealed varying levels of expression of the same gene in leaf and root, respectively (Figure 3(b)). These findings might shed light on the gradual progression of stress response from roots to the leaves of maize.

### 3.2. Gene Set Enrichment and Pathway Analysis

The analysis of gene ontology for biological processes in leaf and root revealed common as well as unique patterns of overexpression. In the leaf, the top five biological processes involved response to oxidation-reduction process (GO: 0055114), inorganic substance (GO: 0010035), abscisic acid (GO: 0009737), abiotic stimulus (GO: 0009628), and temperature stimulus (GO: 0009266) (Figure 4(a)). In the case of root, the top five GO terms denoted response to chemical (GO: 0042221), organic substance (GO: 0010033), oxygen-containing compound (GO: 1901700), lipid (GO: 0033993), and hormone (GO: 0009725) (Figure 4(b)). The pathway enrichment analysis revealed significant expression of the MAPK signaling pathway (KEGG: 04016). The downregulated DEGs did not reveal any significant metabolic pathway. These findings provided an indication of gradual amplification of stress response signatures from roots to the leaves.

### 3.3. PPI Analysis, Hub Genes, and Transcription Factor Identification

The PPI networks determined hub genes with the top five highest degrees. The hub genes commonly expressed in leaves and roots were *low temperature-induced 65* kDa *protein* (LTI65) and *GOLS2*, while those unique to the leaves and roots were *delta-1-pyrroline-5-carboxylate synthase* (P5CS), *ALDH*, *glyoxalase 1* (Glo1), *proline responding 1* (pro1), and *mlg3*, *COR410*, *heat shock protein 70* kDa (HSP70), *beta amylase 1* (BMY1), *heat shock protein 101* kDa (HSP101), *pmpm4*, and *NAC44*, respectively (Figure 5). The *P5CS* and *pro1* identified from leaf and *GOLS2* from root regulated the highest number of genes in their respective networks. On the other hand, the mapping of transcripts to PlantTFDB revealed the presence of several transcription factor families in leaf and root. TFs that were commonly expressed in leaves and roots belonged to the families *NAC*, *HSF*, *bHLH*, *MYB*, and *homeodomain-leucine zipper proteins* (HD-ZIP), respectively. Apart from these, leaves uniquely expressed TFs from *bZIP*, *WRKY*, and *ethylene responsive factor* (*ERF*) families (Table 1).

### 3.4. Transcriptional Regulatory Network in Leaf and Root

Out of 615, 17 DEGs coded for TFs in leaf. A transcriptional regulatory network was constructed taking the identified TFs and DEGs as source and target nodes, respectively. The network did not follow Poisson's distribution (mean = 4.24123; variance = 15,280.46) and hence, the network appeared to display nonrandomness in its distribution. The igraph package in *R* was used to calculate the topological parameters of the network. The network was negatively assortative (-0.2892093), indicating the interaction of nodes with higher degrees with those of smaller ones. This observation was in compliance with real-world biological networks that tend to have negative assortativity [37]. The hub TFs (degree > 4000) identified were *DRE binding factor* (dbf1), *homeobox 41* (hb41), *MYB related 24* (mybr24), *heat shock factor 1* (HSF1), and *heat shock factor 8* (HSF8) (Figure 6(a)).

In the case of root, 7 out of 171 DEGs coded for TFs. The constructed regulatory network for root also did not follow Poisson's distribution (mean = 2.31696; variance = 7,746.27) indicating nonrandomness of the network. The assortativity and degree coefficient of the network were calculated to be -0.0492369 and 7.115, signifying a similar kind of interaction as that of the leaf. The hub TFs (degree > 4000) identified were *NAC44*, *hb41*, and *heat shock factor 17* (*HSF17*) (Figure 6(b)).

## 4. Discussion

### 4.1. Differential Gene Expression of Leaf and Root

This research focused on the study of stress response signatures from protein-coding genes against combined stress of salt and boron in maize. Our analysis showed that the maize plant presents a differential response in leaf and root during combined stress. From our DEG analysis, we found that some genes were completely upregulated in roots. Roots being the primary point of entry of the ions from the soil, an increase in the concentration of sodium and boron ions in soil exhibited overexpression of stress-responsive genes in roots to impart tolerance against the impending stresses.

### 4.2. Gene Set Enrichment and Pathway Analysis

The gene ontology analysis for biological processes indicated a gradient in the amplification of stress resistance from roots to the leaves. The DEGs expressed in roots were concentrated in the response to salt and osmotic stress, oxygen-containing compounds, and hormone as well as in the response to endogenous stimuli of lipid and abscisic acid (Figure 4(a)). The gradient gradually progressed towards leaves that responded to abiotic and chemical stimuli such as osmotic stress, water deprivation, chemical, inorganic substance, and alcohol (Figure 4(b)). This suggested that a major portion of stress tolerance in maize got built up in the root system and progressed towards the shoot system. By comparing the top five GO terms enriched in leaf and root for all three-time points, it was observed that salinity and boron stress were triggered in both leaves and roots for basic metabolic responses. Roots being the first in line for absorption of Na^+^ and B^3+^ initiated the response towards growth and development, while leaves responded in the maintenance of homeostasis and hormone signaling of the system.

As observed from the pathway enrichment analysis, maize exhibited significant upregulation of the secondary metabolite biosynthetic pathway as well as the *mitogen-activated protein kinase* (*MAPK*) signaling pathway. Maize landraces are reported to synthesize a diverse range of secondary metabolites that play important roles throughout the life cycle of the plant. They act as mediators in plant-insect, plant-microorganism, and plant-plant interactions [38]. *MAPKs* are reported to be one of the largest groups of transferase enzymes catalyzing the phosphorylation of protein substrates on serine/threonine residues. They are found in the cytoplasm and nucleus and are involved in the mechanism of signal transduction in plants. By regulating *MAPK* cascades, cells exhibit a wide range of stress responses such as high/low temperature, UV radiation, ozone, ROS, drought, high/low osmolarity, heavy metals, wounding, and pathogen infections. Hormones such as auxin (AUX), abscisic acid (ABA), jasmonic acid (JA), salicylic acid (SA), ethylene (ET), brassinosteroids (BR), and gibberellins/gibberellic acid (GA) have also been shown to enhance their signaling through *MAPK* cascades [39]. These cascades are regulated transcriptionally, translationally, and posttranscriptionally through protein-protein interactions [40], rendering the expression of this pathway an important factor in stress resistance.

### 4.3. Signaling Crosstalk Mechanism between Leaf and Roots against Stress

The overlap of genes of the leaves and roots provided interesting insights into the mechanism of stress resistance in maize. We used UniProt (http://www.uniprot.org) to extract the corresponding proteins of the 94 commonly up-regulated DEGs (Supplementary Table S2) in leaf and root. Both leaf and root were affected differently during combined stress, i.e., root was the primary targeted site through which the ions related to boron as well as salt were accumulated in plants, which gradually affected other parts like leaves. Similar expression of genes in different targeted sites indicates a possible crosstalk mechanism between the leaves and roots in the context of combined stress. Based on their experimentally determined functions, we clustered the significant proteins into five different groups from the commonly expressed 94 proteins (Supplementary Table S2).

One group of proteins was involved in plant defense mechanisms and related processes. During combined stress, the plant becomes weak in its internal environment and needs to protect itself from invading pathogens and their related effectors. Identified proteins like *hexosyltransferase*, *glycosyltransferase* [41], and *chitinase* [42] were involved in the formation of cell walls. Another protein called *12-oxo-phytodienoic acid reductase2* is expressed when a plant receives any physical injury like a wound [43]. This showed that during combined stress, the plant tries to strengthen its first line of defense in both roots and leaves. Apart from this, proteins like *neomenthol dehydrogenase* [44] and *polygalacturonase inhibitors* [45] protect the plant from direct pathogen attack indicating activation of the internal response of the plants against pathogens.

Another group of proteins, the intracellular ABC (ATP-binding cassette) transport family that is known to be involved in the regulation of transport was upregulated, and this group of proteins coordinates the action of the transporters during adverse stress conditions [46]. Other proteins like *phosphatidyl ethanolamine-binding protein* (PEBP) regulate flowering [47] and chloroplast *stay-green protein 1* promote chlorophyll degradation during senescence [48]. Important proteins like *E3 ubiquitin ligase* were involved in protein targeting [48].

Upregulated proteins related to cellular signaling were clustered into a different group. They were involved in calcium ion regulation, transcriptional histone modification signaling, nitrogen and carnitine pathways, and membrane trafficking [49–51]. Calcium is one of the important molecules of plant intracellular signaling that coordinates various intracellular processes, establishing a co-regulatory mechanism between leaf and root [52]. Proteins in this group were also involved in membrane trafficking regulation, a significant defense mechanism during BorSal stress where ion movement within cells is of prime importance [50].

Another set of proteins performs important functions in cellular processes. Proteins like *cytochrome p450* are involved in the cellular metabolism of important xenobiotic substances and are not native to plant systems [53]. Upregulation of *AAA-ATPase* proteins involved in organelle biosynthesis and hypersensitive response helps the plant cope with the combined stress situation [54]. *Nucleus–* and *phragmoplast–localized protein kinase 1* (NPK1) belonging to the *mitogen-associated protein kinase kinase kinase* family (*MAPKKK*) playing a significant role in cell plate formation during the cell cycle [55] is also found to be an overlapping gene between leaf and root. This suggests that the maize plant also establishes coordination between the important cellular processes apart from defense and regulation.

The last group of proteins functions in relation to plant hormones. We observed activation of proteins related to ABA, a master hormone during stress conditions [56]. Proteins related to hormone transport [57] and biosynthesis are expressed along with guard proteins like *nucleoredoxin* which protects antioxidant enzymes from ROS-mediated oxidation [58]. Two putative genes (*Zm00001d038181* and *Zm00001d044529*) coded for the hormone transporter protein family *nitrate transporter 1/peptide transporter* (NRT1/PTR) which plays an important role in the transport of hormones like IAA (indole-3-acetic acid/auxin), GA, JA, and ABA [59]. Genes that coded for hormones were found to express in both leaves and roots, resulting in the coordinated regulation of combined stress. Apart from this, we also observed 8 proteins, which are not yet characterized and have been grouped as unknown (Supplementary Table S2). From the above analysis, it can be inferred that the plant tries to establish complete coordination between root and leaf by activating proteins in both the stress-affected regions.

### 4.4. Protein-Protein Interaction Analysis and Functional Characterization of Hub Genes

As observed from the PPI network analysis, the hub genes identified from leaves are mainly involved in growth and development, regulation of cellular ROS levels coupled with heat shock absorption activity. The genes pro1 and glo1 were reported to play a key role in photorespiratory metabolism which catalyzes the oxidation of glycolate to acetaldehyde and produces H_2_O_2_ (hydrogen peroxide) (Figure 5(a)). The photorespiration process is significant in maize despite it being a C4 plant because of the evolution of this pathway, preventing toxic glycolate accumulation. Hence, C4 photosynthesis in maize is photorespiration-dependent throughout seedling development [60]. The function of *P5CS* was reported in embryogenesis, pollen development, fertility, and reproductive development [61]. Its involvement in proline biosynthesis made it the rate-limiting enzyme and is subjected to feedback inhibition by proline which in turn regulates proline levels under normal as well as in stress conditions [62]. Reports have also shown that proline accumulation increases during salinity stress making it a compatible osmolyte for osmotic adjustment. This phenomenon also results in the stabilization of proteins, membranes, and subcellular structures, buffering of cellular redox potential and protection of cellular functions by ROS [63]. The *aldehyde dehydrogenase* (ALDH) enzyme is reported to be involved in proline homeostasis and indirectly detoxify cellular levels of ROS [64], aids in another development by acting as a nuclear restorer of cytoplasmic male sterility [65]. This enzyme also aids in reducing the effect of cellular toxicity as a result of lipid peroxidation during drought and salt stress [66].

The hub genes from roots are primarily involved in the enhancement of salt tolerance, osmotic stress tolerance, thermotolerance, and regulating membrane potential and cellular ROS production (Figure 5(b)). The *pmpm4*, as its name suggests, is involved in cation uptake that regulates the membrane potential of root cells thereby maintaining intracellular ion homeostasis during salt stress [67]. The *BMY1* and *mlg3* genes function as key regulators of growth and development [68, 69]. While *BMY1* aids in the process of seed germination and maintaining the physiological quality of seeds [70], *mlg3* forms polypeptides that accumulate in the plant tissues as well as in maturing embryos experiencing water deficit. This accumulation of polypeptide is dependent on abscisic acid and limitation of water uptake and helps in protection against osmotic shrinkage during stress [71]. The overexpression of *heat shock proteins* (HSPs) during combined salinity and boron stress gave an indication of the resistant parameters that are induced/coexpressed to confer higher tolerance. *HSP101* is required in the translational enhancement of mRNA [72], for heat-induced thermotolerance [73] and maintenance of a high basal thermotolerant state in the germinating kernels [74]. *HSP70* of the HSP family is involved in regulating cellular ROS levels by maintaining antioxidant enzymes and their activities, resulting in a significant increase in stress tolerance and cryoprotection [75]. It also functions as a chaperone by stabilizing new proteins and ensuring their correct folding or by aiding in the refolding of proteins that were damaged by cell stress [76]. *COR410* belongs to group II *late embryogenesis abundant* (LEA) proteins, also called dehydrins. Dehydrins are the low molecular weight proteins that take part in protective reactions to dehydration in plants [77]. They have been reported to bind to metal ions that can inhibit the production of ROS at its source [78]. They can also bind to DNA as well as proteins and membranes; this would ultimately protect the structural integrity of DNA and the proteins from damage by environmental stress [69].

Apart from the expression of unique hub genes, the two hub genes commonly expressed in the leaves and roots, *LTI65* and *GOLS2*, are involved in response to water deprivation and heat shock promoter activity. LTIs are essentially low-temperature induced ABA-responsive genes [79]. They encode a protein that is expressed in response to water deprivation such as cold, high-salt, and desiccation. This response appears via abscisic acid [80]. *GOLS2* is a key enzyme of the raffinose biosynthetic pathway [81]. The raffinose family of oligosaccharides is essentially *α*-1, 6-galactosyl extensions of sucrose and serves as a desiccation protectant in seeds, protecting the embryo from maturation-associated desiccation [82]. Hence, both leaf and root confer maximum protection in the growth and development of maize seeds.

### 4.5. Identification and Mapping of Transcription Factors

The mapping of transcription factors from PlantTFDB showed the presence of several TF families in the leaves and roots of maize. The transcription factors belonging to the families *NAC*, *HSF*, *bHLH*, *HD-ZIP*, and *MYB* are expressed in both leaves and roots due to their involvement in transcription regulation, DNA binding, regulation of ABA signaling, and cell growth. The *NAC* family is reported to be one of the largest plant-specific transcription factor families that function as positive or negative regulators of plant immunity to biotic stress and as modulators of abiotic stress responses [83]. It has been reported that *NAC44* helps in plant secondary wall formation and in enhancing salt tolerance [84] proving its expression to be of spatial importance against salinity stress response. The HSFs are an important group of stress-responsive TFs identified in a large number of plant species that function in transcriptional regulation during abiotic stress. They have been reported to activate several target genes in response to environmental stresses of high temperature, heavy metals, oxidants, and drought [85]. The *bHLH* transcription factor in plants also comprises one of the largest TF families for their regulation in several abiotic stresses. They regulate their transcriptional expression by specifically binding to the *cis-*elements in the promoter region of the target genes involved in stress response. They also aid in regulating the synthesis of flavonoids that in turn, aid in the maintenance of ROS homeostasis during abiotic stress [86]. HD-ZIP, commonly referred to as *homeodomain-leucine zipper* transcription factors, enhanced tolerance to drought and salt stress and increased sensitivity to abscisic acid [87]. It acts as a positive transcriptional regulator against drought and salt tolerance in plants through an ABA-dependent signaling pathway. The *MYB* transcription factors are found to be widely distributed in plants, mainly for their involvement in ABA response [88] and the phenylpropanoid metabolism pathway. They are directly linked to the control of the cell cycle in plants, thereby promoting maintenance during stress conditions [89]. The transcription factors unique to leaf and roots have shown to be responsive towards different modes of abiotic stresses (Table 1).

### 4.6. Dynamics of Gene Regulatory Network Topology

The hub TFs identified from the regulatory network analysis of leaves were *dbf1*, *hb41*, *mybr24*, *HSF1*, and *HSF8*, and that of roots were *NAC44*, *HSF17*, and *hb41*, respectively.

Studies have shown that dbf1 of the AP2/ERF TF family, also known as *dehydration-responsive element* (DRE) binding factor, is mostly expressed in roots and leaves and involved in the regulation of *rab17*, an ABA responsive gene in an ABA-dependent pathway [106]. The *dbf1* is also reported to be involved in GA signaling resulting in internode development [107] (Figure 6(a)). The overexpression of this TF against osmotic stress in Arabidopsis conferred more tolerance than control plants. It also acts as a transcriptional activator by binding to the GCC-box, an ethylene-responsive element that promotes upregulation of pathogenesis-related (PR) genes in the event of pathogen attack [108].

The TF *hb41* shows expression in leaves, roots, and panicles of maize during drought stress, which is an indicator of change in water potential of leaves due to applied salinity stress. It regulates sodium-hydrogen pumps ((*AtNHX1/AtNHX6*)-*Arabidopsis thaliana* vacuolar Na^+^/H^+^ antiporter) that balances cellular Na^+^ ions. *hb41* activates the enzymes superoxide dismutase (SOD) and peroxidase (POD) that help the plant in ROS scavenging [86] (Figure 6(b)).

The *mybr24* belongs to the MYB TF family that plays a significant role in plant defense against various stress conditions. It activates late stamen development by ubiquitylating the *Jasmonate Zim* domain (JAZ) through a JA-mediated signaling pathway [109]. MYB TFs are reported to be involved in the negative regulation of salt-sensitive overlay (SOS) protein which is a sodium/potassium transporter [110] and activation of the ABA signaling pathway [111] conferring stress tolerance.

*HSF1* and *HSF8* of the HSF family are known for epigenetic regulation by binding to a number of genes such as *APX2 (ascorbate peroxidase*), HSPs, *heat stress-associated 32-kD* protein (Hsa32), *FK506-binding proteins* (ROF1), and *drought-regulated gene 29A* (RD29A) which coordinately help the plant in stress adaptation [112]. *NAC44*, a part of the NAC TF family (NAM, ATAF1, 2, and CUC2), is involved in drought and salt tolerance, growth, and development [113, 117] by regulating defense responsive genes like *late embryogenesis abundant* (LEA), *glutathione S-transferase* (GST), and *glyoxalase*.

*GRAS* (*Gibberellic-Acid Insensitive* (GAI), *Repressor of GAI* (Rga), and *Scarecrow* (SCR)) is another large TF family divided into 8 major subfamilies. They are involved in root and shoot development, GA signaling, and phytochrome A signal transduction [114]. In our study, a *GRAS* TF is found to be significantly upregulated, and it is known to aid in stress tolerance by regulating seed germination, hypocotyl cell elongation, and root elongation [115].

**Figure 7:**
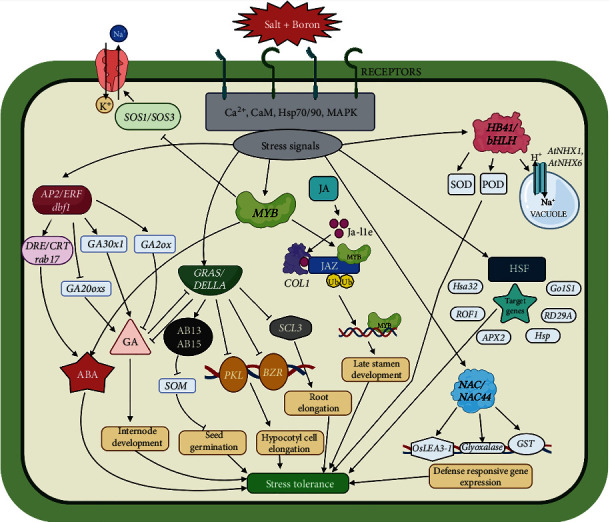
Overview of the transcriptional regulatory mechanism in maize in response to combined salinity and boron stress. (SOS: salt sensitive overlay; *CaM*: *calmodulin*; HSP: heat shock protein; *MAPK*: *mitogen associated protein kinase*; *HB41*: *homeobox 41*; *bHLH*: *basic helix loop helix*; SOD: superoxide dismutase; POD: peroxidase; *AtNHX1/6*: *Arabidopsis thaliana* sodium hydrogen antiporter; *AP2/ERF*: apetala2/ethylene responsive factor; *dbf1*: *DRE binding factor*; *DRE/CRT*: drought response element/C-repeat; GA20oxs: gibberelin-20 oxidase; ABA: abscisic acid; GA: gibberelic acid; *GRAS*: (*Gibberellic-Acid Insensitive* (GAI), *Repressor of GAI* (Rga), and *Scarecrow (*SCR)); *SOM*: *SOMNUS*; *SCL3*: scarecrow-like 3; *PKL*: *PICKLE*; *BZR*: *brassinosteroid-resistant*; JA: jasmonic acid; *JAZ*: *jasmonate zim* domain; *COL1*: *CONSTANS-like 1*; JA-Ile: jasmonic acid induced isoleucine; HSF: heat shock factor; *hsa32*: *heat stress-associated 32*; *ROF1*: *rotamase FKBP 1*; APX2: ascorbate peroxidase 2; *Gols1*: *Galactinol synthase 1*; *NAC*: (*NAM* (*no apical meristem, Petunia*), *ATAF1–2 (Arabidopsis thaliana activating factor*), and *CUC2* (*cup-shaped cotyledon, Arabidopsis*)); *OsLEA3-1*: *Oryza sativa late embryogenesis abundant 3-1*; GST: glutathione S-transferase).

As observed from the regulatory network topology, it can be inferred that upon being subjected to combined salinity and boron stress in maize, a number of key transcription factors contribute to the overall growth and regulation by enhancing individual as well as combined tolerance to stress (Figure 7) (created using BioRender [116]).

## 5. Conclusions

From our analysis, we observed that response against combined salinity and boron stress in maize occurs uniformly from the roots, exhibiting expression of functionally similar genes throughout the plant to gradually diverging into an organ-specific response. Our results have elucidated that the roots of maize protect the plant from water deprivation and desiccation while the leaves aid in maintaining proper growth and development during stress conditions. Our observations have also predicted the possible activation of a signal crosstalk mechanism between the leaves and roots with an overlapping expression of genes and transcription factors regulating defense response, hormonal response, and cellular processes. Further study on the significant hub genes and transcription factors would help understand the mechanistic insights of their working and to develop multistress-resistant varieties of maize.

## Figures and Tables

**Table 1:** Common and unique transcription factor families identified in leaf and root of maize using command line-based mapping of DEGs to Plant TFDB.

Sl. No.	TF ID	TF family	TF common name	Found in (leaf/root/both)	Function	References
1	*GRMZM2G002131_P01*	HSF	*HSF1*	Leaf	Transcription regulation, sequence-specific DNA binding	[90, 91]
2	*GRMZM2G011598_P01*	NAC	*NAC44*	Both	Transcription regulation, DNA binding	[33]
3	*GRMZM2G014653_P01*	NAC	*LOC101027155*	Leaf	Response to wounding, negative regulation of abscisic acid-activated signaling pathway	
4	*GRMZM2G045883_P01*	bHLH	*bHLH161*	Leaf	Protein dimerization activity	
5	*GRMZM2G092137_P01*	bZIP	*bZIP9*	Leaf	Transcription factor activity, sequence-specific DNA binding	
6	*GRMZM2G105348_P01*	HSF	*Zm.96358*	Both	Transcription factor activity, sequence-specific DNA binding	[90]
7	*GRMZM2G109627_P01*	NAC	*NAC118*	Leaf	Multidimensional cell growth, fruit ripening, flower development, leaf senescence	[33]
8	*GRMZM2G118047_P01*	HSF	*LOC10120269*	Leaf	Transcription factor activity, sequence-specific DNA binding	[92]
9	*GRMZM2G127379_P01*	NAC	*LOC100502408*	Leaf	Stress-induced transcription factor NAC1	[93–95]
10	*GRMZM2G164909_P01*	HSF	*LOC100286024*	Leaf	Response to chitin	[90, 92, 96]
11	*GRMZM2G180328_P01*	NAC	*NAC20, SNAC052*	Both	Regulation of transcription, DNA-templated	[33, 90]
12	*GRMZM2G336533_P01*	NAC	*pco133091*	Leaf	Regulation of transcription, DNA-templated	[91, 97, 98]
13	*GRMZM2G347043_P01*	NAC	*NAC49, SNAC1*	Leaf	Regulation of transcription, DNA-templated	[90, 98, 99]
14	*GRMZM5G858197_P02*	bZIP	*Zm.81747*	Leaf	Transcription factor activity, sequence-specific DNA binding	[90]
15	*GRMZM5G871347_P03*	WRKY	*Zm.86376*	Leaf	Regulation of transcription, DNA-templated, integral component of membrane	[100, 101]
16	*GRMZM2G061487_P01*	ERF	*EREB20*	Leaf	Transcription factor activity, sequence-specific DNA binding	[33]
17	*GRMZM2G171179_P01*	ERF	*EREB160, umc1588*	Leaf	Transcription factor activity, sequence-specific DNA binding	[102–104]
18	*GRMZM2G351330_P01*	HD-ZIP	*HB66*	Both	Transcription factor activity, sequence-specific DNA binding	[33]
19	*GRMZM2G117164_P01*	HD-ZIP	*Zm.18537*	Both	Transcription factor activity, sequence-specific DNA binding	[33]
20	*GRMZM2G396527_P01*	HD-ZIP	*HB70*	Both	Transcription factor activity, sequence-specific DNA binding	[33]
21	*GRMZM2G143640_P01*	MYB_Related	*LOC103636453*	Both	Regulation of transcription, DNA-templated	[105]

## Data Availability

The data used to support the findings of this study are included within the supplementary information file(s).

## Supplementary Materials

Supplementary Materials Supplementary Table S1: list of differentially expressed genes in maize leaf and root in response to combined salt and boron stress. Supplementary Table S2: functions of the commonly upregulated DEGs in leaf and root derived from UniProt.

## References

[1] Laborde D., Martin W., Swinnen J., McDermott J. (2020). Poverty and food insecurity could grow dramatically as COVID-19 spreads. *COVID-19 and global food security*.

[2] von Grebmer K., Bernstein J., Delgado C. (2021). Figure 1.6: 2021 Global hunger index by severity. *Map in 2021 Global Hunger Index: Hunger and Food Systems in Conflict Settings*.

[3] Bushra P., Sharma P. (2020). Climate change and its impacts on Indian agriculture: an econometric analysis. *Journal of Public Affairs*.

[4] Luck J., Spackman M., Freeman A. (2011). Climate change and diseases of food crops. *Plant Pathology*.

[5] Nobuhiro S., Rivero R. M., Shulaev V., Blumwald E., Mittler R. (2014). Abiotic and biotic stress combinations. *New Phytologist*.

[6] Pandey A., Khan M. K., Hakki E. E., Gezgin S., Hamurcu M. (2019). Combined boron toxicity and salinity stress—an insight into its interaction in plants. *Plants*.

[7] Parida A. K., Das A. B. (2005). Salt tolerance and salinity effects on plants: a review. *Ecotoxicology and Environmental Safety*.

[8] Munns R., Tester M. (2008). Mechanisms of salinity tolerance. *Annual Review of Plant Biology*.

[9] Isayenkov S. V., Maathuis F. J. (2019). Plant salinity stress: many unanswered questions remain. *Frontiers in Plant Science*.

[10] Princi M. P., Lupini A., Araniti F., Ahmad P. (2016). Boron toxicity and tolerance in plants. *Plant Metal Interaction*.

[11] Nable R. O., Bañuelos G. S., Paull J. G. (1997). Boron toxicity. *Plant and Soil*.

[12] Broadley M., Brown P., Cakmak I., Rengel Z., Zhao F., Marchner P. (2012). Function of nutrients: micronutrients. *Marschner’s mkeral nutrition of higher plants*.

[13] Barah P., Bone A. M. (2015). Multidimensional approaches for studying plant defence against insects: from ecology to omics and synthetic biology. *Journal of Experimental Botany*.

[14] Sahu A., Das A., Saikia K., Barah P. (2020). Temperature differentially modulates the transcriptome response in *Oryza sativa* to *Xanthomonas oryzae pv. oryzae* infection. *Genomics*.

[15] Zandalinas S. I., Fichman Y., Devireddy A. R., Sengupta S., Azad R. K., Mittler R. (2020). Systemic signaling during abiotic stress combination in plants. *Proceedings of the National Academy of Sciences*.

[16] Qian Y., Ren Q., Zhang J., Chen L. (2019). Transcriptomic analysis of the maize (*Zea mays* L.) inbred line B73 response to heat stress at the seedling stage. *Gene*.

[17] Chen F., Gao J., Li W., Fang P. (2022). Transcriptome profiles reveal the protective role of seed coating with zinc against boron toxicity in maize (*Zea mays* L.). *Journal of Hazardous Materials*.

[18] Nawaz M., Ishaq S., Ishaq H. (2020). Salicylic acid improves boron toxicity tolerance by modulating the physio-biochemical characteristics of maize (Zea mays L.) at an early growth stage. *Agronomy*.

[19] Li P., Cao W., Fang H. (2017). Transcriptomic profiling of the maize (*Zea mays* L.) leaf response to abiotic stresses at the seedling stage. *Frontiers in Plant Science*.

[20] Guo Q., Li X., Niu L., Jameson P. E., Zhou W. (2021). Transcription-associated metabolomic adjustments in maize occur during combined drought and cold stress. *Plant Physiology*.

[21] Li Y., Wang X., Li Y. (2020). Transcriptomic analysis revealed the common and divergent responses of maize seedling leaves to cold and heat stresses. *Genes*.

[22] Li H., Yue H., Xie J. (2021). Transcriptomic profiling of the high-vigour maize (*Zea mays* L.) hybrid variety response to cold and drought stresses during seed germination. *Scientific Reports*.

[23] Huanca-Mamani W., Arias-Carrasco R., Cárdenas-Ninasivincha S. (2018). Long non-coding RNAs responsive to salt and boron stress in the hyper-arid Lluteño maize from Atacama Desert. *Genes*.

[24] Andrews S. FastQC: A Quality Control Tool for High Throughput Sequencing. https://www.bioinformatics.babraham.ac.uk/projects/fastqc/.

[25] Chen S., Zhou Y., Chen Y., Gu J. (2018). fastp: an ultra-fast all-in-one FASTQ preprocessor. *Bioinformatics*.

[26] Jiao Y., Peluso P., Shi J. (2017). Improved maize reference genome with single-molecule technologies. *Nature*.

[27] Liao Y., Smyth G., Shi W. (2014). featureCounts: an efficient general purpose program for assigning sequence reads to genomic features. *Bioinformatics*.

[28] Love M., Huber W., Anders S. (2014). Moderated estimation of fold change and dispersion for RNA-seq data with DESeq2. *Genome Biology*.

[29] Howe E., Holton K., Nair S., Schlauch D., Sinha R., Quackenbush J., Ochs M., Casagrande J., Davuluri R. (2010). MeV: multiexperiment viewer. *Biomedical Informatics for Cancer Research*.

[30] Raudvere U., Kolberg L., Kuzmin I. (2019). g:Profiler: a web server for functional enrichment analysis and conversions of gene lists (2019 update). *Nucleic Acid Research*.

[31] Mering C. V., Huynen M., Jaeggi D., Schmidt S., Bork P., Snel B. (2003). STRING: a database of predicted functional associations between proteins. *Nucleic Acid Research*.

[32] Shannon P., Markiel A., Ozier O. (2003). Cytoscape: a software environment for integrated models of biomolecular interaction networks. *Genome Research*.

[33] Jin J., Tian F., Yang D. C. (2017). PlantTFDB 4.0: toward a central hub for transcription factors and regulatory interactions in plants. *Nucleic Acids Research*.

[34] Weirauch M. T., Yang A., Albu M. (2014). Determination and inference of eukaryotic transcription factor sequence specificity. *Cell*.

[35] Thomas-Chollier M., Sand O., Turatsinze J. V. (2008). RSAT: regulatory sequence analysis tools. *Nucleic Acids Research*.

[36] Grant C. E., Bailey T. L., Noble W. S. (2011). FIMO: scanning for occurrences of a given motif. *Bioinformatics*.

[37] Barabási A. L. (2009). Scale-free networks: a decade and beyond. *Science*.

[38] Uarrota V. G., Severino R. B., Maraschin M. (2011). Maize landraces (*Zea mays* L.): a new prospective source for secondary metabolite production. *International Journal of Agricultural Research*.

[39] Jagodzik P., Tajdel-Zielinska M., Ciesla A., Marczak M., Ludwikow A. (2018). Mitogen-activated protein kinase cascades in plant hormone signaling. *Frontiers in Plant Science*.

[40] Taj G., Agarwal P., Grant M., Kumar A. (2010). MAPK machinery in plants. *Plant signalling & behaviour*.

[41] Hansen S. F., Harholt J., Oikawa A., Scheller H. V. (2012). Plant glycosyltransferases beyond CAZy: a perspective on DUF families. *Frontiers in Plant Science*.

[42] Kumar M., Brar A., Yadav M., Chawade A., Vivekanand V., Pareek N. (2018). Chitinases—potential candidates for enhanced plant resistance towards fungal pathogens. *Agriculture*.

[43] Taki N., Sasaki-Sekimoto Y., Obayashi T. (2005). 12-oxo-phytodienoic acid triggers expression of a distinct set of genes and plays a role in wound-induced gene expression in Arabidopsis. *Plant Physiology*.

[44] Choi H. W., Lee B. G., Kim N. H. (2008). A role for a menthone reductase in resistance against microbial pathogens in plants. *Plant Physiology*.

[45] Liu N., Zhang X., Sun Y. (2017). Molecular evidence for the involvement of a polygalacturonase-inhibiting protein, GhPGIP1, in enhanced resistance to Verticillium and Fusarium wilts in cotton. *Scientific Reports*.

[46] Kang J., Park J., Choi H. (2011). Plant ABC transporters. *The Arabidopsis book/American Society of Plant Biologists*.

[47] Liu Y., Yang K., Wei X., Wang X. (2016). Revisiting the phosphatidylethanolamine-binding protein (PEBP) gene family reveals cryptic FLOWERING LOCUS T gene homologs in gymnosperms and sheds new light on functional evolution. *New Phytologist*.

[48] Barry C. S., McQuinn R. P., Chung M.-Y., Besuden A., Giovannoni J. J. (2008). Amino acid substitutions in homologs of the STAY-GREEN protein are responsible for thegreen-fleshandchlorophyll retainermutations of tomato and pepper. *Plant Physiology*.

[49] Lelandais-Brière C., Jovanovic M., Torres G. A. M. (2007). Disruption of AtOCT1, an organic cation transporter gene, affects root development and carnitine-related responses in Arabidopsis. *The Plant Journal*.

[50] Petersen N. H. T., Joensen J., McKinney L. V. (2009). Identification of proteins interacting with *Arabidopsis* ACD11. *Journal of Plant Physiology*.

[51] Hu R., Xiao J., Gu T. (2018). Genome-wide identification and analysis of WD40 proteins in wheat (Triticum aestivum L.). *BMC Genomics*.

[52] Chen L., Hellmann H. (2013). Plant E3 ligases: flexible enzymes in a sessile world. *Molecular Plant*.

[53] Manikandan P., Nagini S. (2018). Cytochrome P450 structure, function and clinical significance: a review. *Current Drug Targets*.

[54] Baek K., Seo P. J., Park C.-M. (2011). Activation of a mitochondrial ATPase gene induces abnormal seed development in Arabidopsis. *Molecules and Cells*.

[55] Nishihama R., Ishikawa M., Araki S., Soyano T., Asada T., Machida Y. (2001). The NPK1 mitogen-activated protein kinase kinase kinase is a regulator of cell-plate formation in plant cytokinesis. *Genes & Development*.

[56] Guo W.-J., David Ho T.-H. (2008). An abscisic acid-induced protein, HVA22, inhibits gibberellin-mediated programmed cell death in cereal Aleurone cells. *Plant Physiology*.

[57] Chiba Y., Shimizu T., Miyakawa S. (2015). Identification of Arabidopsis thaliana NRT1/PTR FAMILY (NPF) proteins capable of transporting plant hormones. *Journal of Plant Research*.

[58] Kneeshaw S., Keyani R., Delorme-Hinoux V. (2017). Nucleoredoxin guards against oxidative stress by protecting antioxidant enzymes. *Proceedings of the National Academy of Sciences*.

[59] Corratgé-Faillie C., Lacombe B. (2017). Substrate (un)specificity of Arabidopsis NRT1/PTR FAMILY (NPF) proteins. *Journal of Experimental Botany*.

[60] Zelitch I., Schultes N. P., Peterson R. B., Brown P., Brutnell T. P. (2009). High glycolate oxidase activity is required for survival of maize in normal air. *Plant Physiology*.

[61] Trovato M., Mattioli R., Costantino P. (2018). From a. rhizogenes RolD to plant P5CS: exploiting proline to control plant development. *Plants*.

[62] Yang S., Lan S., Gong M. (2009). Hydrogen peroxide-induced proline and metabolic pathway of its accumulation in maize seedlings. *Journal of Plant Physiology*.

[63] Špoljarević M., Agić D., Lisjak M. (2011). The relationship of proline content and metabolism on the productivity of maize plants. *Plant Signalling & Behaviour*.

[64] Liu F., Schnable P. S. (2002). Functional specialization of maize mitochondrial aldehyde dehydrogenases. *Plant Physiology*.

[65] Jimenez-Lopez J. C., Gachomo E. W., Seufferheld M. J., Kotchoni S. O. (2010). The maize ALDH protein superfamily: linking structural features to functional specificities. *BMC Structural Biology*.

[66] Tola A., Jaballi A., Germain H., Missihoun T. (2020). Recent development on plant aldehyde dehydrogenase enzymes and their functions in plant development and stress signaling. *Genes*.

[67] Opitz N., Marcon C., Paschold A. (2016). Extensive tissue-specific transcriptomic plasticity in maize primary roots upon water deficit. *Journal of Experimental Botany*.

[68] Oliveira G., Pinho R., Andrade T., Pinho É. V. . R. V., Santos C. D. ., Veiga A. D. (2013). Physiological quality and amylase enzyme expression in maize seeds. *Ciência e Agrotecnologia*.

[69] Amara I., Odena A., Oliveira E. (2012). Insights into maize LEA proteins: from proteomics to functional approaches. *Plant and Cell Physiology*.

[70] Miao M., Li R., Huang C., Jiang B., Zhang T. (2015). Impact of *β*-amylase degradation on properties of sugary maize soluble starch particles. *Food Chemistry*.

[71] Thomann E., Sollinger J., White C., Rivin C. J. (1992). Accumulation of group 3 late embryogenesis abundant proteins inzea maysembryos. *Plant Physiology*.

[72] Young T., Ling J., Geisler-Lee C., Tanguay R. L., Caldwell C., Gallie D. R. (2001). Developmental and thermal regulation of the maize heat shock protein, HSP101. *Plant Physiology*.

[73] Nieto-Sotelo J., Martínez L. M., Ponce G. (2002). Maize HSP101 plays important roles in both induced and basal thermotolerance and primary root growth. *The Plant Cell*.

[74] Lázaro-Mixteco P. E., Nieto-Sotelo J., Swatek K. N. (2012). The absence of heat shock protein HSP101 affects the proteome of mature and germinating maize embryos. *Journal of Proteome Reaserch*.

[75] Abou-Deif M. H., Rashed M. A.-S., Khalil K. M., Mahmoud F. E.-S. (2019). Proteomic analysis of heat shock proteins in maize (Zea mays L.). *Bulletin of the National Research Centre*.

[76] Hu X., Liu R., Li Y. (2010). Heat shock protein 70 regulates the abscisic acid-induced antioxidant response of maize to combined drought and heat stress. *Plant Growth Regulation*.

[77] Liu Y., Song Q., Li D., Yang X., Li D. (2017). Multifunctional roles of plant dehydrins in response to environmental stresses. *Frontiers in Plant Science*.

[78] Koag M., Fenton R., Wilkens S., Close T. J. (2003). The binding of maize DHN1 to lipid vesicles. Gain of structure and lipid specificity. *Plant Physiology*.

[79] Msanne J., Lin J., Stone J. M., Awada T. (2011). Characterization of abiotic stress-responsive Arabidopsis thaliana RD29A and RD29B genes and evaluation of transgenes. *Planta*.

[80] Nordin K., Vahala T., Palva E. T. (1993). Differential expression of two related, low-temperature-induced genes in Arabidopsis thaliana (L.) Heynh. *Plant Molecular Biology*.

[81] Gu L., Han Z., Zhang L., Downie B., Zhao T. (2013). Functional analysis of the 5′ regulatory region of the maize *GALACTINOL SYNTHASE2* gene. *Plant Science*.

[82] Sengupta S., Mukherjee S., Basak P., Majumder A. L. (2015). Significance of galactinol and raffinose family oligosaccharide synthesis in plants. *Frontiers in Plant Science*.

[83] Li X., Jiang Y. (2021). Expression of ZmNAC3 responsive to various abiotic stresses in maize (Zea mays L.). *Bangladesh Journal of Botany*.

[84] Wang G., Yuan Z., Zhang P., Liu Z., Wang T., Wei L. (2020). Genome-wide analysis of NAC transcription factor family in maize under drought stress and rewatering. *Physiology and Molecular Biology of Plants*.

[85] Zhang H., Li G., Fu C., Duan S., Hu D., Guo X. (2020). Genome-wide identification, transcriptome analysis and alternative splicing events of Hsf family genes in maize. *Scientific Reports*.

[86] Qian Y., Zhang T., Yu Y. (2021). Regulatory mechanisms of bHLH transcription factors in plant adaptive responses to various abiotic stresses. *Frontiers in Plant Science*.

[87] Zhao Y., Ma Q., Jin X. (2014). A novel maize Homeodomain–Leucine zipper (HD-zip) I gene, Zmhdz10, positively regulates drought and salt tolerance in both rice and Arabidopsis. *Plant & Cell Physiology*.

[88] Ambawat S., Sharma P., Yadav N. R., Yadav R. C. (2013). MYB transcription factor genes as regulators for plant responses: an overview. *Physiology and Molecular Biology of Plants*.

[89] Du H., Feng B.-R., Yang S.-S., Huang Y.-B., Tang Y.-X. (2012). The R2R3-MYB transcription factor gene family in maize. *PLoS One*.

[90] Kikuchi S., Satoh K., Nagata T. (2003). Collection, mapping, and annotation of over 28,000 cDNA clones from *japonica* Rice. *Science*.

[91] Grand X., Espinoza R., Michel C. (2012). Identification of positive and negative regulators of disease resistance to rice blast fungus using constitutive gene expression patterns. *Plant Biotechnology Journal*.

[92] Alexandrov N. N., Brover V. V., Freidin S. (2009). Insights into corn genes derived from large-scale cDNA sequencing. *Plant Molecular Biology*.

[93] The Tomato Genome Consortium (2012). The tomato genome sequence provides insights into fleshy fruit evolution. *Nature*.

[94] Mao H., Wang H., Liu S. (2015). A transposable element in a *NAC* gene is associated with drought tolerance in maize seedlings. *Nature Communications*.

[95] Ma X., Zhang Y., Turečková V. (2018). The NAC transcription factor SlNAP2 regulates leaf senescence and fruit yield in tomato. *Plant Physiology*.

[96] Xiang J., Ran J., Zou J. (2013). Heat shock factor OsHsfB2b negatively regulates drought and salt tolerance in rice. *Plant Cell Reports*.

[97] Chen Q., Wang Q., Xiong L., Lou Z. (2011). A structural view of the conserved domain of rice stress-responsive NAC1. *Protein & Cell*.

[98] Li J., Guo G., Guo W. (2012). miRNA164-directed cleavage of ZmNAC1 confers lateral root development in maize (*Zea mays* L.). *BMC Plant Biology*.

[99] Lu M., Ying S., Zhang D. F. (2012). A maize stress-responsive NAC transcription factor, ZmSNAC1, confers enhanced tolerance to dehydration in transgenic Arabidopsis. *Plant Cell Reports*.

[100] Chai J., Liu J., Zhou J., Xing D. (2014). Mitogen-activated protein kinase 6 regulates NPR1 gene expression and activation during leaf senescence induced by salicylic acid. *Journal of Experimental Botany*.

[101] Zhou F., Menke F. L. H., Yoshioka K., Moder W., Shirano Y., Klessig D. F. (2004). High humidity suppresses ssi4-mediated cell death and disease resistance upstream of MAP kinase activation, H2O2 production and defense gene expression. *The Plant Journal*.

[102] Kim C. Y., Lee S. H., Park H. C. (2000). Identification of rice blast fungal elicitor-responsive genes by differential display analysis. *Molecular Plant-Microbe Interactions*.

[103] Koo S. C., Moon B. C., Kim J. K. (2009). OsBWMK1 mediates SA-dependent defense responses by activating the transcription factor OsWRKY33. *Biochemical and Biophysical Research Communications*.

[104] Iwamoto M., Takano M. (2011). Phytochrome-regulated EBL1 contributes to ACO1 upregulation in rice. *Biotechnology Letters*.

[105] Almeida J., Rocheta M., Galego L. (1997). Genetic control of flower shape in Antirrhinum majus. *Development*.

[106] Saleh A., Lumbreras V., Lopez C., Dominguez-Puigjaner E., Kizis D., Pagès M. (2006). Maize DBF1-interactor protein 1 containing an R3H domain is a potential regulator of DBF1 activity in stress responses. *The Plant Journal*.

[107] Xie Z., Nolan T. M., Jiang H., Yin Y. (2019). AP2/ERF transcription factor regulatory networks in hormone and abiotic stress responses in Arabidopsis. *Frontiers in Plant Science*.

[108] Okamuro J. K., Caster B., Villarroel R., van Montagu M., Jofuku K. D. (1997). The AP2 domain of *APETALA2* defines a large new family of DNA binding proteins in Arabidopsis. *Proceedings of the National Academy of Sciences of the United States of America*.

[109] Song S., Qi T., Huang H., Xie D. (2013). Regulation of stamen development by coordinated actions of jasmonate, auxin, and gibberellin in *Arabidopsis*. *Molecular Plant*.

[110] Kim J., Nguyen N., Jeong C., Nguyen N. T., Hong S. W., Lee H. (2013). Loss of the R2R3 MYB, AtMyb73, causes hyper-induction of the *SOS1* and *SOS3* genes in response to high salinity in *Arabidopsis*. *Journal of Plant Physiology*.

[111] Li J., Han G., Sun C., Sui N. (2019). Research advances of MYB transcription factors in plant stress resistance and breeding. *Taylor & Francis*.

[112] Guo M., Liu J. H., Ma X., Luo D. X., Gong Z. H., Lu M. H. (2016). The plant heat stress transcription factors (HSFS): structure, regulation, and function in response to abiotic stresses. *Frontiers in Plant Science*.

[113] Yuan X., Wang H., Cai J., Li D., Song F. (2019). NAC transcription factors in plant immunity. *Phytopathology Research*.

[114] Hirsch S., Oldroyd G. E. D. (2009). GRAS-domain transcription factors that regulate plant development. *Plant Signaling and Behavior*.

[115] Kumari P., Kakkar M., Gahlaut V., Jaiswal V., Kumar S. Multifarious roles of GRAS transcription factors in plants. https://www.preprints.org/manuscript/202103.0066/v1.

[116] Gallery. https://app.biorender.com/.

[117] Takasaki H., Maruyama K., Kidokoro S. (2010). The abiotic stress-responsive NAC-type transcription factor OsNAC5 regulates stress-inducible genes and stress tolerance in rice. *Molecular Genetics and Genomics*.

