# Supplementary material for: Identifying Signal-Crosstalk Mechanism in Maize Plants during Combined Salinity and Boron Stress Using Integrative Systems Biology Approaches
Source: Biomed Res Int. 2022 Apr 18;2022:1027288. doi: 10.1155/2022/1027288 (PMC9057046; doi:10.1155/2022/1027288)
Supplement: Supplementary Materials — Supplementary Table S1: list of differentially expressed genes in maize leaf and root in response to combined salt and boron stress. Supplementary Table S2: functions of the commonly upregulated DEGs in leaf and root derived from UniProt. [file 1027288.f1.docx]

**Identifying signal-crosstalk mechanism in Maize plants during combined salinity and boron stress using integrative systems biology approaches**

**Authors:**Drishtee Barua^1^, Asutosh Mishra^1^, P.B. Kirti^2^, and Pankaj Barah^1*^

1. Department of Molecular Biology and Biotechnology, Tezpur University, Assam, 784028, India

2. Agri Biotech Foundation, Rajendranagar, Agricultural University Campus, Hyderabad, 500030, India,

*Corresponding authors: Pankaj Barah: [barah@tezu.ernet.in](mailto:barah@tezu.ernet.in)

Manuscript Submitted to BioMed Research International (Manuscript ID: 1027288)

**Supplementary Materials:**

**1. Supplementary table S1:** List of differentially expressed genes in maize leaf and root in response to combined salinity and boron stress.

**(A)** Differentially expressed genes in leaves of maize (up-regulated) in response to combined salt and boron stress

**(B)** Differentially expressed genes in leaves of maize (down-regulated) in response to combined salinity and boron stress

**(C)** List of Differentially expressed genes in roots of maize (up-regulated) in response to combined salinity and boron stress

**2. Supplementary table S2:** Functions of the commonly upregulated DEGs in leaf and root derived from UniProt.

**1. Supplementary table S1:** List of differentially expressed genes in maize leaf and root in response to combined salinity and boron stress.

1. *Differentially expressed genes in leaves of maize (up-regulated) in response to combined salt and boron stress*

| **Gene ID** | **Log2FC** | **3 h** | **96 h** | **0 h** |
| --- | --- | --- | --- | --- |
| *Zm00001d011231* | 2.498502767 | 10.96984539 | 10.85268173 | 8.5303939 |
| *Zm00001d027625* | 2.538635097 | 11.49434432 | 11.86096154 | 9.222989669 |
| *Zm00001d003265* | 2.560935899 | 11.96478636 | 12.15313715 | 9.55849229 |
| *Zm00001d045475* | 2.61082427 | 9.892255557 | 9.526417978 | 7.377076293 |
| *Zm00001d029620* | 2.612855369 | 10.0725949 | 10.8342255 | 8.056260409 |
| *Zm00001d039052* | 2.634570374 | 9.308124192 | 9.608770133 | 7.148208519 |
| *Zm00001d012338* | 2.682495224 | 9.661553744 | 9.698537195 | 7.286202437 |
| *Zm00001d031013* | 2.710238 | 9.366650583 | 10.28315459 | 7.445778452 |
| *Zm00001d034188* | 2.715506619 | 9.965338681 | 10.8723337 | 7.954500012 |
| *Zm00001d022243* | 2.749957989 | 9.356493673 | 9.357023884 | 6.971751402 |
| *Zm00001d016176* | 2.757178814 | 8.789212595 | 9.236356722 | 6.713861017 |
| *Zm00001d045044* | 2.759326662 | 9.775176665 | 9.245214094 | 7.106145971 |
| *Zm00001d040670* | 2.761475003 | 9.406576907 | 10.4538578 | 7.511293255 |
| *Zm00001d016299* | 2.767426879 | 9.416387959 | 10.01984634 | 7.276783576 |
| *Zm00001d020686* | 2.779208524 | 10.67676512 | 9.545660521 | 7.662932649 |
| *Zm00001d034617* | 2.785842538 | 8.919205482 | 9.622426937 | 6.911667502 |
| *Zm00001d039512* | 2.79757676 | 8.6955264 | 9.131970158 | 6.610719812 |
| *Zm00001d009700* | 2.800774761 | 10.99296332 | 9.832832493 | 7.912881914 |
| *Zm00001d039851* | 2.804929792 | 10.1012502 | 9.191233794 | 7.218903334 |
| *Zm00001d042609* | 2.811669377 | 9.980843508 | 10.47595862 | 7.662932649 |
| *Zm00001d044024* | 2.821856379 | 9.915494498 | 9.144651033 | 7.095429172 |
| *Zm00001d051056* | 2.830787816 | 12.22306527 | 12.49484197 | 9.592619975 |
| *Zm00001d010056* | 2.832513582 | 9.71286678 | 9.648246188 | 7.168774149 |
| *Zm00001d020315* | 2.839579372 | 10.18586239 | 9.860943295 | 7.454135803 |
| *Zm00001d033483* | 2.840567286 | 12.98628919 | 12.30686738 | 9.892632527 |
| *Zm00001d049597* | 2.845752653 | 10.33635155 | 9.962849535 | 7.558512274 |
| *Zm00001d033273* | 2.853773089 | 11.60351045 | 11.85783946 | 8.972314641 |
| *Zm00001d038866* | 2.85645649 | 10.88882151 | 10.4940176 | 8.023139062 |
| *Zm00001d035139* | 2.857513913 | 10.30853744 | 9.875271083 | 7.503267982 |
| *Zm00001d052061* | 2.881271788 | 9.279737284 | 8.826247393 | 6.655888039 |
| *Zm00001d034757* | 2.886767368 | 8.424017682 | 9.459509183 | 6.625944278 |
| *Zm00001d004564* | 2.89111599 | 11.04655914 | 10.48843725 | 8.072537686 |
| *Zm00001d008727* | 2.9087888 | 12.53235271 | 11.64195194 | 9.316740888 |
| *Zm00001d014191* | 2.912027477 | 8.737920135 | 8.930658015 | 6.465326001 |
| *Zm00001d049054* | 2.91274188 | 8.819132188 | 8.657201519 | 6.395236226 |
| *Zm00001d032295* | 2.918758976 | 10.18777223 | 8.914082806 | 7.106145971 |
| *Zm00001d024963* | 2.923938999 | 9.1757308 | 8.429708234 | 6.465326001 |
| *Zm00001d028230* | 2.933632894 | 9.820202837 | 10.46904907 | 7.503267982 |
| *Zm00001d022594* | 2.938353825 | 8.524382771 | 9.450603824 | 6.610719812 |
| *Zm00001d002601* | 2.983358738 | 10.23471428 | 9.807155042 | 7.341427677 |
| *Zm00001d006034* | 2.987495706 | 8.309078193 | 8.600740101 | 6.159140144 |
| *Zm00001d033797* | 2.990244085 | 10.20673281 | 10.8541288 | 7.786809268 |
| *Zm00001d020025* | 2.990589859 | 10.06222127 | 10.35714825 | 7.487079551 |
| *Zm00001d038167* | 3.000986096 | 9.136686739 | 9.941221638 | 6.971751402 |
| *Zm00001d046364* | 3.002394294 | 7.876820029 | 8.685707488 | 6.069677158 |
| *Zm00001d012398* | 3.019940327 | 8.222012143 | 9.510591968 | 6.515566756 |
| *Zm00001d042869* | 3.020173003 | 11.79292158 | 12.4709922 | 9.227825038 |
| *Zm00001d031290* | 3.030793076 | 9.268946277 | 8.7720776 | 6.531901887 |
| *Zm00001d043735* | 3.052101847 | 8.629494766 | 10.43593001 | 7.095429172 |
| *Zm00001d047290* | 3.057194889 | 9.017075546 | 8.165471934 | 6.242718449 |
| *Zm00001d008808* | 3.070937604 | 8.937502325 | 8.473155846 | 6.282555675 |
| *Zm00001d042536* | 3.074487027 | 9.268946277 | 9.386675716 | 6.713861017 |
| *Zm00001d047045* | 3.075115839 | 8.462481667 | 9.256939817 | 6.413110089 |
| *Zm00001d043083* | 3.086208482 | 10.20673281 | 10.47721135 | 7.51927327 |
| *Zm00001d037239* | 3.095193868 | 11.0359981 | 10.77942848 | 8.000623865 |
| *Zm00001d035475* | 3.095979961 | 9.863865137 | 10.96587538 | 7.655723576 |
| *Zm00001d026070* | 3.10498953 | 9.322110572 | 9.834788866 | 6.899330622 |
| *Zm00001d009146* | 3.113026866 | 12.3538169 | 10.00862581 | 8.617830569 |
| *Zm00001d021006* | 3.128287736 | 8.055614689 | 8.199388423 | 5.868711397 |
| *Zm00001d015829* | 3.133438741 | 10.26397459 | 10.20591389 | 7.385849289 |
| *Zm00001d007085* | 3.135850454 | 8.748326063 | 8.503051697 | 6.180552184 |
| *Zm00001d023979* | 3.138159515 | 9.544178439 | 8.71152812 | 6.563986152 |
| *Zm00001d009858* | 3.139456769 | 10.86515155 | 11.35598698 | 8.156353298 |
| *Zm00001d013033* | 3.141057424 | 10.68354787 | 11.84431272 | 8.377504174 |
| *Zm00001d037934* | 3.149684784 | 10.26939541 | 10.2534843 | 7.394568031 |
| *Zm00001d029673* | 3.15026254 | 10.38377163 | 10.32202106 | 7.470703652 |
| *Zm00001d012515* | 3.152220074 | 8.706242625 | 8.582193514 | 6.180552184 |
| *Zm00001d020345* | 3.158637273 | 11.99781206 | 11.17947558 | 8.606723127 |
| *Zm00001d051872* | 3.176051284 | 10.76893844 | 9.415729524 | 7.359364751 |
| *Zm00001d002690* | 3.176606524 | 12.38494168 | 12.68286412 | 9.432247596 |
| *Zm00001d026670* | 3.189820588 | 7.638970353 | 8.349671866 | 5.783781739 |
| *Zm00001d029550* | 3.21084008 | 11.03705769 | 9.630334049 | 7.542946898 |
| *Zm00001d048234* | 3.2207097 | 7.715658072 | 8.643851745 | 5.895723542 |
| *Zm00001d002940* | 3.227940981 | 9.899266656 | 8.690043268 | 6.67061512 |
| *Zm00001d052977* | 3.230716388 | 8.357526812 | 8.259069274 | 5.922142194 |
| *Zm00001d003671* | 3.245272743 | 7.484519917 | 8.072000806 | 5.62671642 |
| *Zm00001d015356* | 3.248747882 | 9.790342194 | 11.61972234 | 7.924897152 |
| *Zm00001d037684* | 3.249820766 | 8.244279651 | 8.034884596 | 5.812776776 |
| *Zm00001d051163* | 3.252876125 | 9.213744055 | 8.028031416 | 6.180552184 |
| *Zm00001d028428* | 3.25585577 | 9.906243824 | 8.939784618 | 6.713861017 |
| *Zm00001d018856* | 3.256028087 | 8.589526765 | 7.355812206 | 5.783781739 |
| *Zm00001d039194* | 3.256468361 | 9.29400067 | 10.03778935 | 6.874320638 |
| *Zm00001d022391* | 3.276060357 | 8.316100469 | 8.000285226 | 5.812776776 |
| *Zm00001d040029* | 3.280985409 | 8.629494766 | 9.249622435 | 6.321205767 |
| *Zm00001d024732* | 3.284486655 | 7.497017515 | 7.644088067 | 5.482757748 |
| *Zm00001d038540* | 3.302798462 | 7.828387571 | 7.764736811 | 5.592483533 |
| *Zm00001d006329* | 3.31099628 | 8.946564227 | 9.763658326 | 6.610719812 |
| *Zm00001d049954* | 3.314301069 | 9.633794769 | 10.19224703 | 7.006588491 |
| *Zm00001d040028* | 3.315568097 | 9.390075203 | 9.563469986 | 6.655888039 |
| *Zm00001d049107* | 3.320646398 | 7.672344661 | 8.344173903 | 5.723527321 |
| *Zm00001d038267* | 3.328192075 | 8.462481667 | 9.537273508 | 6.377116502 |
| *Zm00001d007446* | 3.348865457 | 12.17670841 | 10.87376119 | 8.45391841 |
| *Zm00001d034978* | 3.353783922 | 11.69898135 | 10.97610103 | 8.191533473 |
| *Zm00001d038165* | 3.355159664 | 9.206221258 | 9.295823817 | 6.465326001 |
| *Zm00001d011193* | 3.363715558 | 9.092478812 | 9.721982909 | 6.595321515 |
| *Zm00001d047554* | 3.366179277 | 7.638970353 | 8.870835422 | 5.895723542 |
| *Zm00001d003304* | 3.368827129 | 8.843598315 | 8.098401836 | 5.973313413 |
| *Zm00001d028998* | 3.368912987 | 7.353056763 | 9.343341333 | 6.069677158 |
| *Zm00001d023799* | 3.369628439 | 8.350705002 | 8.140290683 | 5.812776776 |
| *Zm00001d005798* | 3.370334059 | 8.19177316 | 8.101667961 | 5.754047241 |
| *Zm00001d006900* | 3.380314964 | 11.89987509 | 9.823995808 | 8.011925812 |
| *Zm00001d040002* | 3.382781957 | 8.651844142 | 9.880015542 | 6.548040015 |
| *Zm00001d022492* | 3.393472577 | 10.08903902 | 10.67522103 | 7.323259112 |
| *Zm00001d038768* | 3.394809889 | 8.251625897 | 9.343341333 | 6.20161093 |
| *Zm00001d003309* | 3.407794249 | 8.6955264 | 10.84978325 | 7.095429172 |
| *Zm00001d025964* | 3.408240658 | 8.991036983 | 8.614494575 | 6.137361376 |
| *Zm00001d028968* | 3.411119059 | 7.895741546 | 8.666032963 | 5.84107402 |
| *Zm00001d050347* | 3.416061296 | 9.825120024 | 9.002099664 | 6.579745111 |
| *Zm00001d014953* | 3.419705372 | 8.727438336 | 10.41058186 | 6.822894158 |
| *Zm00001d035501* | 3.422899833 | 9.550131381 | 9.305735807 | 6.548040015 |
| *Zm00001d033633* | 3.42298734 | 8.456142169 | 7.216835796 | 5.62671642 |
| *Zm00001d039065* | 3.431648082 | 9.672509344 | 8.867012605 | 6.465326001 |
| *Zm00001d031677* | 3.432977303 | 8.566178492 | 10.23508842 | 6.685183962 |
| *Zm00001d027652* | 3.435526133 | 13.05798124 | 11.5797581 | 9.14844039 |
| *Zm00001d028992* | 3.440852625 | 10.87229341 | 10.00515569 | 7.359364751 |
| *Zm00001d045387* | 3.447780457 | 8.030181053 | 8.174801854 | 5.692171077 |
| *Zm00001d046952* | 3.461279978 | 9.920097671 | 10.79156301 | 7.276783576 |
| *Zm00001d042886* | 3.470898551 | 9.224955074 | 8.310735904 | 6.115201596 |
| *Zm00001d051803* | 3.481517418 | 8.129308978 | 7.748115468 | 5.592483533 |
| *Zm00001d002958* | 3.483251561 | 8.57790007 | 7.089469227 | 5.62671642 |
| *Zm00001d047787* | 3.495792196 | 7.977914104 | 7.946762228 | 5.592483533 |
| *Zm00001d028759* | 3.502737137 | 8.499941681 | 7.701379243 | 5.692171077 |
| *Zm00001d005826* | 3.507604919 | 8.236895542 | 8.81834986 | 5.922142194 |
| *Zm00001d048201* | 3.509574259 | 13.08666189 | 12.13579254 | 9.256501198 |
| *Zm00001d052060* | 3.532416297 | 10.1012502 | 9.430039623 | 6.741946189 |
| *Zm00001d014135* | 3.533689075 | 7.569770586 | 7.293815515 | 5.315569993 |
| *Zm00001d033718* | 3.553361382 | 8.047186992 | 8.855482817 | 5.868711397 |
| *Zm00001d032536* | 3.556469617 | 8.716879547 | 10.17690815 | 6.595321515 |
| *Zm00001d043382* | 3.565409594 | 10.917873 | 12.40407872 | 8.420457916 |
| *Zm00001d004705* | 3.568067656 | 9.764976941 | 9.294402233 | 6.531901887 |
| *Zm00001d044529* | 3.573639311 | 8.343850585 | 7.234978895 | 5.520602495 |
| *Zm00001d019563* | 3.6043923 | 12.09563585 | 11.36852896 | 8.324201812 |
| *Zm00001d004248* | 3.607109615 | 9.038420177 | 10.11225508 | 6.595321515 |
| *Zm00001d017340* | 3.622026415 | 8.105163642 | 7.739731411 | 5.520602495 |
| *Zm00001d029560* | 3.624809127 | 8.706242625 | 7.621422532 | 5.692171077 |
| *Zm00001d027601* | 3.63801958 | 8.336963246 | 6.911893389 | 5.443487124 |
| *Zm00001d030577* | 3.644366805 | 7.161446385 | 7.81346715 | 5.315569993 |
| *Zm00001d002614* | 3.660089288 | 9.770085824 | 10.83910535 | 7.106145971 |
| *Zm00001d020963* | 3.668656495 | 9.439023885 | 9.90164117 | 6.563986152 |
| *Zm00001d014664* | 3.671554107 | 7.533861284 | 8.187147912 | 5.482757748 |
| *Zm00001d007097* | 3.677372878 | 8.862875914 | 7.607647379 | 5.723527321 |
| *Zm00001d047418* | 3.689517139 | 8.481333867 | 11.22939078 | 7.106145971 |
| *Zm00001d034501* | 3.693657021 | 9.854276045 | 9.908159009 | 6.685183962 |
| *Zm00001d052357* | 3.704062472 | 6.920665824 | 8.282262468 | 5.402648995 |
| *Zm00001d029716* | 3.704516535 | 7.238269593 | 8.778198226 | 5.62671642 |
| *Zm00001d043263* | 3.709007741 | 9.464465402 | 7.67081754 | 5.973313413 |
| *Zm00001d018966* | 3.71037737 | 9.132723462 | 10.50142464 | 6.741946189 |
| *Zm00001d026649* | 3.710427459 | 7.282420737 | 7.825393587 | 5.315569993 |
| *Zm00001d011782* | 3.72046619 | 11.31378277 | 9.333685627 | 7.248141901 |
| *Zm00001d047913* | 3.723346702 | 8.748326063 | 8.672621111 | 5.895723542 |
| *Zm00001d027520* | 3.723352028 | 7.366768318 | 7.38851224 | 5.219736028 |
| *Zm00001d043853* | 3.724508916 | 7.011523434 | 9.962849535 | 6.159140144 |
| *Zm00001d033872* | 3.735446409 | 10.27479593 | 11.49634655 | 7.542946898 |
| *Zm00001d048901* | 3.765544136 | 8.251625897 | 8.966822153 | 5.84107402 |
| *Zm00001d040190* | 3.767691562 | 13.1230393 | 12.28575604 | 9.085890824 |
| *Zm00001d050196* | 3.769384212 | 8.236895542 | 7.234978895 | 5.402648995 |
| *Zm00001d018699* | 3.76997575 | 9.217490767 | 8.338654778 | 5.947996523 |
| *Zm00001d042948* | 3.781875779 | 7.704952835 | 8.475671086 | 5.557142754 |
| *Zm00001d022132* | 3.797567923 | 10.62414654 | 7.964827045 | 6.579745111 |
| *Zm00001d026662* | 3.80067751 | 7.886312167 | 8.609924367 | 5.62671642 |
| *Zm00001d027649* | 3.804230959 | 7.828387571 | 9.469619791 | 5.947996523 |
| *Zm00001d025786* | 3.817347165 | 8.57790007 | 7.621422532 | 5.557142754 |
| *Zm00001d034601* | 3.848063732 | 11.61064502 | 11.45339616 | 7.894667177 |
| *Zm00001d029151* | 3.850406785 | 12.8217436 | 11.0261694 | 8.474441853 |
| *Zm00001d026368* | 3.852891572 | 8.824058823 | 8.836058712 | 5.895723542 |
| *Zm00001d051403* | 3.854993462 | 9.969785672 | 9.311369327 | 6.430745199 |
| *Zm00001d030498* | 3.863199647 | 8.168667336 | 7.957628524 | 5.482757748 |
| *Zm00001d051917* | 3.87332726 | 6.507158786 | 8.068666142 | 5.219736028 |
| *Zm00001d052767* | 3.873550239 | 8.055614689 | 8.713659048 | 5.65992188 |
| *Zm00001d043515* | 3.874078632 | 6.605302343 | 8.570479102 | 5.402648995 |
| *Zm00001d042721* | 3.874741828 | 8.506091071 | 8.579858262 | 5.723527321 |
| *Zm00001d022367* | 3.888829427 | 6.803098555 | 7.975557238 | 5.219736028 |
| *Zm00001d033931* | 3.890922091 | 7.161446385 | 9.382668044 | 5.783781739 |
| *Zm00001d016982* | 3.899756541 | 9.12076775 | 8.577519208 | 5.895723542 |
| *Zm00001d006509* | 3.900984328 | 8.236895542 | 8.395572656 | 5.592483533 |
| *Zm00001d018964* | 3.907415694 | 6.993822424 | 7.492343358 | 5.112423372 |
| *Zm00001d019565* | 3.915005534 | 8.54841478 | 7.714278579 | 5.520602495 |
| *Zm00001d026632* | 3.91749697 | 8.623852678 | 7.789310443 | 5.557142754 |
| *Zm00001d052018* | 3.9176224 | 7.932847912 | 8.124324392 | 5.443487124 |
| *Zm00001d020612* | 3.940731808 | 12.01296908 | 10.43914779 | 7.726219365 |
| *Zm00001d014617* | 3.949497075 | 9.104670772 | 6.751300776 | 5.592483533 |
| *Zm00001d052253* | 3.952443356 | 6.920665824 | 8.788341727 | 5.482757748 |
| *Zm00001d040726* | 3.963947809 | 7.353056763 | 8.544366052 | 5.443487124 |
| *Zm00001d018965* | 3.985772979 | 8.168667336 | 9.318380383 | 5.84107402 |
| *Zm00001d042143* | 3.987780586 | 6.605302343 | 9.080099305 | 5.557142754 |
| *Zm00001d048021* | 3.998012548 | 9.715517519 | 9.392001995 | 6.262790439 |
| *Zm00001d044285* | 3.99879175 | 7.161446385 | 8.541968424 | 5.402648995 |
| *Zm00001d032138* | 4.006303464 | 6.455245719 | 7.666397417 | 5.053155642 |
| *Zm00001d027375* | 4.018331471 | 8.055614689 | 7.59373714 | 5.315569993 |
| *Zm00001d009631* | 4.020814803 | 12.0199525 | 10.25713548 | 7.626515568 |
| *Zm00001d007079* | 4.032580516 | 7.569770586 | 8.3982275 | 5.402648995 |
| *Zm00001d014097* | 4.038172716 | 7.886312167 | 8.196338091 | 5.402648995 |
| *Zm00001d052435* | 4.038406499 | 7.867264283 | 10.99546343 | 6.640999032 |
| *Zm00001d038358* | 4.044543916 | 7.420318478 | 7.829346982 | 5.219736028 |
| *Zm00001d002799* | 4.044984633 | 9.183414155 | 9.980628205 | 6.282555675 |
| *Zm00001d037689* | 4.050203502 | 6.69678093 | 7.833289439 | 5.112423372 |
| *Zm00001d044301* | 4.056531179 | 10.67812422 | 10.91778832 | 7.116781043 |
| *Zm00001d053212* | 4.074402595 | 7.113241841 | 9.155655915 | 5.592483533 |
| *Zm00001d045888* | 4.083364954 | 7.282420737 | 7.522061379 | 5.112423372 |
| *Zm00001d029356* | 4.107968588 | 7.545932483 | 6.457514637 | 4.989073369 |
| *Zm00001d047582* | 4.11005945 | 12.38909669 | 9.7071071 | 7.726219365 |
| *Zm00001d002610* | 4.12927081 | 8.047186992 | 8.208500626 | 5.402648995 |
| *Zm00001d042088* | 4.130714977 | 7.096796458 | 9.71986717 | 5.812776776 |
| *Zm00001d020627* | 4.144792006 | 7.995549745 | 6.598164637 | 5.112423372 |
| *Zm00001d039914* | 4.153206542 | 8.294929589 | 9.336451011 | 5.783781739 |
| *Zm00001d007789* | 4.157868603 | 6.651814267 | 7.492343358 | 4.989073369 |
| *Zm00001d022155* | 4.159239679 | 8.206972463 | 9.305735807 | 5.754047241 |
| *Zm00001d013934* | 4.160966126 | 6.344881788 | 9.298662781 | 5.557142754 |
| *Zm00001d011202* | 4.191706276 | 9.656044556 | 9.094877873 | 6.046278952 |
| *Zm00001d045455* | 4.198499658 | 7.72628331 | 7.81346715 | 5.219736028 |
| *Zm00001d017852* | 4.208388985 | 8.343850585 | 8.232520151 | 5.443487124 |
| *Zm00001d038408* | 4.251302459 | 9.579532715 | 8.3982275 | 5.84107402 |
| *Zm00001d018122* | 4.264876953 | 7.282420737 | 7.160952266 | 4.989073369 |
| *Zm00001d012510* | 4.292860375 | 7.757690923 | 9.402595837 | 5.65992188 |
| *Zm00001d039081* | 4.326938336 | 10.34322206 | 10.26004978 | 6.548040015 |
| *Zm00001d017240* | 4.328886368 | 7.960057963 | 10.67248955 | 6.262790439 |
| *Zm00001d049860* | 4.333773129 | 7.238269593 | 7.906196043 | 5.112423372 |
| *Zm00001d052079* | 4.336466654 | 10.24575658 | 12.07752473 | 7.403233202 |
| *Zm00001d047778* | 4.338005464 | 6.975892528 | 6.68138413 | 4.840849447 |
| *Zm00001d032186* | 4.352777708 | 7.778250684 | 8.411428518 | 5.315569993 |
| *Zm00001d005818* | 4.360216724 | 10.82031104 | 13.06915686 | 8.16143228 |
| *Zm00001d006499* | 4.374575432 | 7.60479244 | 9.280108693 | 5.557142754 |
| *Zm00001d052443* | 4.380376013 | 6.920665824 | 6.809670312 | 4.840849447 |
| *Zm00001d017288* | 4.383126177 | 10.13133325 | 9.284411669 | 6.159140144 |
| *Zm00001d052194* | 4.385645899 | 7.096796458 | 9.230421398 | 5.482757748 |
| *Zm00001d047548* | 4.387227402 | 5.902250225 | 8.217555218 | 5.053155642 |
| *Zm00001d029654* | 4.401661135 | 9.221227757 | 9.455699338 | 5.895723542 |
| *Zm00001d011131* | 4.415203874 | 8.424017682 | 7.906196043 | 5.315569993 |
| *Zm00001d017434* | 4.418047203 | 7.296834626 | 6.335502905 | 4.840849447 |
| *Zm00001d027755* | 4.424719428 | 8.176410786 | 8.579858262 | 5.402648995 |
| *Zm00001d022069* | 4.432448501 | 8.063992995 | 8.117887655 | 5.268888732 |
| *Zm00001d042826* | 4.434820058 | 9.458147015 | 5.845391089 | 5.482757748 |
| *Zm00001d028307* | 4.446548072 | 6.019120965 | 8.447758004 | 5.112423372 |
| *Zm00001d028180* | 4.469676507 | 11.62762538 | 13.6593392 | 8.632508158 |
| *Zm00001d052375* | 4.471767097 | 6.901755281 | 7.40458215 | 4.918936994 |
| *Zm00001d024598* | 4.473233555 | 6.40121846 | 7.954015607 | 4.989073369 |
| *Zm00001d045370* | 4.476369877 | 8.804250202 | 6.064911484 | 5.219736028 |
| *Zm00001d047663* | 4.476847123 | 6.882581836 | 8.25614355 | 5.112423372 |
| *Zm00001d052961* | 4.47905812 | 6.557127854 | 7.607647379 | 4.918936994 |
| *Zm00001d010798* | 4.480309982 | 7.986759142 | 6.369916291 | 4.989073369 |
| *Zm00001d033229* | 4.480475874 | 7.969013943 | 6.425342136 | 4.989073369 |
| *Zm00001d048969* | 4.486615557 | 6.091303685 | 7.456864163 | 4.840849447 |
| *Zm00001d029087* | 4.491372709 | 9.733937444 | 11.26681231 | 6.713861017 |
| *Zm00001d043988* | 4.493880636 | 8.012970317 | 11.81538785 | 6.874320638 |
| *Zm00001d033455* | 4.498119201 | 6.507158786 | 9.354297751 | 5.443487124 |
| *Zm00001d047799* | 4.528333452 | 6.344881788 | 8.468112105 | 5.112423372 |
| *Zm00001d043293* | 4.536281754 | 7.459188346 | 6.263827215 | 4.840849447 |
| *Zm00001d011080* | 4.53936242 | 8.973412467 | 12.84360936 | 7.655723576 |
| *Zm00001d053818* | 4.554125863 | 6.224339817 | 7.477246888 | 4.840849447 |
| *Zm00001d038846* | 4.554922585 | 11.44968768 | 11.17755048 | 7.137811086 |
| *Zm00001d039575* | 4.572064383 | 7.339210402 | 9.969097038 | 5.723527321 |
| *Zm00001d040500* | 4.573311035 | 8.229473173 | 7.240974797 | 5.112423372 |
| *Zm00001d031267* | 4.582862766 | 9.108711921 | 10.97654398 | 6.395236226 |
| *Zm00001d004547* | 4.585060454 | 7.446350222 | 8.121109643 | 5.112423372 |
| *Zm00001d011132* | 4.596596633 | 9.373382288 | 8.891681226 | 5.692171077 |
| *Zm00001d011839* | 4.614283746 | 6.428507921 | 6.978470465 | 4.751626261 |
| *Zm00001d018179* | 4.621054909 | 7.459188346 | 8.589176576 | 5.219736028 |
| *Zm00001d049061* | 4.642075383 | 7.046262483 | 7.07606933 | 4.840849447 |
| *Zm00001d028720* | 4.650846001 | 7.080154741 | 9.195810247 | 5.360076373 |
| *Zm00001d003190* | 4.650872293 | 7.627668935 | 10.43141303 | 5.922142194 |
| *Zm00001d021885* | 4.662254137 | 8.54841478 | 7.252890259 | 5.167740011 |
| *Zm00001d024004* | 4.670878458 | 9.209987569 | 10.66316364 | 6.20161093 |
| *Zm00001d033503* | 4.673552876 | 6.019120965 | 8.259069274 | 4.989073369 |
| *Zm00001d038806* | 4.675030041 | 8.153053632 | 9.984157782 | 5.754047241 |
| *Zm00001d009497* | 4.676285155 | 9.268946277 | 9.417036331 | 5.754047241 |
| *Zm00001d028293* | 4.67698396 | 8.14518244 | 9.115960368 | 5.443487124 |
| *Zm00001d014258* | 4.700005019 | 10.3415075 | 11.5217589 | 6.822894158 |
| *Zm00001d016255* | 4.709314102 | 9.744358386 | 9.881908963 | 5.998117711 |
| *Zm00001d016166* | 4.710043892 | 10.08699373 | 11.22417788 | 6.610719812 |
| *Zm00001d019936* | 4.718762346 | 10.79673151 | 10.99415149 | 6.699598117 |
| *Zm00001d037724* | 4.744323306 | 8.236895542 | 6.478537551 | 4.989073369 |
| *Zm00001d029091* | 4.746561087 | 9.526170624 | 11.22827531 | 6.482284377 |
| *Zm00001d020317* | 4.751427129 | 6.920665824 | 6.742754963 | 4.751626261 |
| *Zm00001d014703* | 4.752155415 | 7.208044088 | 7.082785391 | 4.840849447 |
| *Zm00001d025665* | 4.810069427 | 8.606792241 | 9.839668215 | 5.692171077 |
| *Zm00001d026253* | 4.835818496 | 10.07672355 | 8.270712794 | 5.723527321 |
| *Zm00001d038181* | 4.83640623 | 7.85764406 | 9.852277768 | 5.592483533 |
| *Zm00001d002847* | 4.842575671 | 7.046262483 | 10.84007934 | 5.998117711 |
| *Zm00001d024778* | 4.843091038 | 11.20768032 | 10.31573234 | 6.563986152 |
| *Zm00001d024486* | 4.843248537 | 7.253141489 | 8.551535011 | 5.112423372 |
| *Zm00001d020638* | 4.872087552 | 7.080154741 | 8.149785646 | 4.989073369 |
| *Zm00001d047555* | 4.880782996 | 10.09922217 | 9.653798382 | 5.947996523 |
| *Zm00001d023384* | 4.88638964 | 6.192360529 | 7.837221017 | 4.840849447 |
| *Zm00001d039566* | 4.897719106 | 6.628758477 | 8.798414175 | 5.112423372 |
| *Zm00001d045361* | 4.912393345 | 6.255542868 | 7.845051784 | 4.840849447 |
| *Zm00001d007753* | 4.920804061 | 8.222012143 | 11.83603774 | 6.595321515 |
| *Zm00001d030542* | 4.932248535 | 5.817538239 | 8.290864048 | 4.918936994 |
| *Zm00001d039769* | 4.971541784 | 8.905328514 | 10.01726472 | 5.723527321 |
| *Zm00001d044421* | 4.976816515 | 6.557127854 | 8.897314408 | 5.112423372 |
| *Zm00001d042114* | 4.977812038 | 8.727438336 | 8.121109643 | 5.219736028 |
| *Zm00001d016664* | 4.983869777 | 11.52864835 | 11.93050829 | 7.137811086 |
| *Zm00001d012420* | 4.987102704 | 9.356493673 | 11.22976241 | 6.302024259 |
| *Zm00001d039416* | 4.995454455 | 6.993822424 | 8.335887228 | 4.989073369 |
| *Zm00001d030544* | 5.007152959 | 5.772790879 | 8.628118613 | 4.989073369 |
| *Zm00001d011236* | 5.0513151 | 7.161446385 | 7.57024579 | 4.840849447 |
| *Zm00001d043536* | 5.085642956 | 8.391157461 | 7.154602919 | 4.989073369 |
| *Zm00001d010007* | 5.088844728 | 8.19177316 | 6.881241085 | 4.918936994 |
| *Zm00001d040067* | 5.115681321 | 7.177151997 | 7.648578066 | 4.840849447 |
| *Zm00001d010652* | 5.116050069 | 7.818500387 | 5.575471164 | 4.751626261 |
| *Zm00001d027619* | 5.119012754 | 12.64817117 | 11.70398424 | 7.437371358 |
| *Zm00001d020631* | 5.121938891 | 7.85764406 | 8.625856892 | 5.112423372 |
| *Zm00001d043795* | 5.123762379 | 8.475077272 | 8.095128237 | 5.112423372 |
| *Zm00001d036532* | 5.140382824 | 7.788420268 | 7.653053911 | 4.918936994 |
| *Zm00001d029313* | 5.142994247 | 6.605302343 | 6.776621911 | 4.64507569 |
| *Zm00001d046299* | 5.148151931 | 7.683297405 | 8.49315543 | 5.053155642 |
| *Zm00001d030540* | 5.151279138 | 5.726251078 | 7.805460355 | 4.751626261 |
| *Zm00001d009084* | 5.160541694 | 8.168667336 | 9.154088929 | 5.268888732 |
| *Zm00001d033357* | 5.166806744 | 7.995549745 | 6.66332816 | 4.840849447 |
| *Zm00001d013220* | 5.169331972 | 9.004115152 | 8.950660672 | 5.360076373 |
| *Zm00001d008573* | 5.180838279 | 7.029001539 | 6.335502905 | 4.64507569 |
| *Zm00001d042975* | 5.183018957 | 7.22323831 | 5.944347934 | 4.64507569 |
| *Zm00001d010743* | 5.19177386 | 7.747298572 | 7.198454713 | 4.840849447 |
| *Zm00001d038221* | 5.193462984 | 7.960057963 | 9.111122507 | 5.219736028 |
| *Zm00001d002261* | 5.206738986 | 6.651814267 | 9.123987516 | 5.112423372 |
| *Zm00001d026460* | 5.2074582 | 7.380347725 | 7.625984689 | 4.840849447 |
| *Zm00001d014325* | 5.213674556 | 9.15243164 | 11.22939078 | 6.137361376 |
| *Zm00001d043992* | 5.213744295 | 9.625361738 | 9.277232884 | 5.557142754 |
| *Zm00001d016070* | 5.227062702 | 7.253141489 | 10.54269413 | 5.65992188 |
| *Zm00001d013830* | 5.23351594 | 6.055734554 | 8.226552927 | 4.840849447 |
| *Zm00001d044142* | 5.237082636 | 7.253141489 | 6.00610174 | 4.64507569 |
| *Zm00001d012604* | 5.240446206 | 6.40121846 | 8.149785646 | 4.840849447 |
| *Zm00001d021288* | 5.259737967 | 7.080154741 | 8.616774239 | 4.989073369 |
| *Zm00001d029769* | 5.266088286 | 6.863137744 | 6.699203027 | 4.64507569 |
| *Zm00001d023294* | 5.276912994 | 6.605302343 | 6.949275613 | 4.64507569 |
| *Zm00001d042880* | 5.289420241 | 8.690138176 | 10.25713548 | 5.62671642 |
| *Zm00001d031736* | 5.300736385 | 6.019120965 | 7.293815515 | 4.64507569 |
| *Zm00001d044078* | 5.301984738 | 10.62837115 | 9.287273194 | 5.84107402 |
| *Zm00001d038049* | 5.305803042 | 8.030181053 | 9.228933737 | 5.219736028 |
| *Zm00001d014794* | 5.314770879 | 8.977838874 | 7.679616736 | 5.112423372 |
| *Zm00001d027443* | 5.314909626 | 8.302021348 | 7.864442521 | 4.989073369 |
| *Zm00001d026223* | 5.323955333 | 8.206972463 | 6.559463526 | 4.840849447 |
| *Zm00001d014072* | 5.341355337 | 7.080154741 | 6.549611477 | 4.64507569 |
| *Zm00001d017682* | 5.354221379 | 8.121305724 | 8.802423483 | 5.112423372 |
| *Zm00001d000294* | 5.358418505 | 8.872418686 | 8.728487741 | 5.219736028 |
| *Zm00001d017453* | 5.365624909 | 7.238269593 | 6.335502905 | 4.64507569 |
| *Zm00001d047736* | 5.368954222 | 8.662889814 | 8.945232921 | 5.219736028 |
| *Zm00001d027946* | 5.374302753 | 6.192360529 | 7.299565859 | 4.64507569 |
| *Zm00001d047993* | 5.383914565 | 8.960050963 | 8.349671866 | 5.167740011 |
| *Zm00001d008795* | 5.398585293 | 6.939320872 | 8.81834986 | 4.989073369 |
| *Zm00001d040011* | 5.404436289 | 8.323088514 | 6.457514637 | 4.840849447 |
| *Zm00001d037005* | 5.427229578 | 5.817538239 | 8.460513104 | 4.840849447 |
| *Zm00001d045063* | 5.431554882 | 6.557127854 | 7.173565109 | 4.64507569 |
| *Zm00001d037453* | 5.448128634 | 10.19917847 | 12.04706219 | 6.548040015 |
| *Zm00001d039310* | 5.448822004 | 5.726251078 | 8.091847128 | 4.751626261 |
| *Zm00001d051843* | 5.45645516 | 6.939320872 | 6.904293953 | 4.64507569 |
| *Zm00001d018429* | 5.484132357 | 9.084293095 | 10.36731902 | 5.62671642 |
| *Zm00001d018819* | 5.4894464 | 8.716879547 | 6.626477589 | 4.918936994 |
| *Zm00001d020780* | 5.511785756 | 7.208044088 | 9.854207947 | 5.268888732 |
| *Zm00001d018298* | 5.565867707 | 8.113257449 | 9.533664077 | 5.219736028 |
| *Zm00001d029366* | 5.606617594 | 10.97427746 | 8.661624026 | 5.754047241 |
| *Zm00001d024522* | 5.659880314 | 7.55790167 | 6.347074794 | 4.64507569 |
| *Zm00001d048085* | 5.715667895 | 7.113241841 | 7.122416011 | 4.64507569 |
| *Zm00001d014947* | 5.716016855 | 11.30852735 | 8.926991102 | 5.868711397 |
| *Zm00001d047399* | 5.728193577 | 6.993822424 | 7.246945177 | 4.64507569 |
| *Zm00001d033878* | 5.74595233 | 7.638970353 | 7.58438726 | 4.751626261 |
| *Zm00001d037610* | 5.767239912 | 9.084293095 | 7.517151516 | 4.989073369 |
| *Zm00001d039757* | 5.77113617 | 8.499941681 | 4.697786404 | 4.751626261 |
| *Zm00001d021628* | 5.781849124 | 8.371073845 | 7.492343358 | 4.840849447 |
| *Zm00001d007180* | 5.795697243 | 5.57391538 | 7.891153994 | 4.64507569 |
| *Zm00001d041472* | 5.884901424 | 7.046262483 | 8.180988191 | 4.751626261 |
| *Zm00001d028303* | 5.927659757 | 8.999768954 | 11.13016193 | 5.692171077 |
| *Zm00001d025533* | 5.962336003 | 5.459064121 | 9.017696729 | 4.840849447 |
| *Zm00001d002592* | 5.971631452 | 6.76155977 | 7.709991792 | 4.64507569 |
| *Zm00001d004138* | 5.993530457 | 7.986759142 | 6.107268634 | 4.64507569 |
| *Zm00001d012322* | 5.995774605 | 5.902250225 | 8.021145214 | 4.64507569 |
| *Zm00001d029738* | 6.011369621 | 6.507158786 | 6.690322801 | 4.505258212 |
| *Zm00001d008983* | 6.032471356 | 11.54661567 | 10.32202106 | 5.973313413 |
| *Zm00001d033300* | 6.033941439 | 8.891316388 | 10.83520279 | 5.520602495 |
| *Zm00001d029164* | 6.050534536 | 7.471910876 | 7.288041547 | 4.64507569 |
| *Zm00001d042780* | 6.062393735 | 5.677739507 | 8.127531935 | 4.64507569 |
| *Zm00001d049288* | 6.070907316 | 6.224339817 | 6.956632259 | 4.505258212 |
| *Zm00001d048333* | 6.103927939 | 6.40121846 | 9.389341317 | 4.918936994 |
| *Zm00001d007901* | 6.124689302 | 11.89870982 | 9.7657108 | 5.998117711 |
| *Zm00001d019078* | 6.165382587 | 9.675235282 | 10.60189115 | 5.482757748 |
| *Zm00001d016705* | 6.212912171 | 6.315778261 | 7.062539241 | 4.505258212 |
| *Zm00001d018064* | 6.222084852 | 4.759138418 | 8.366040405 | 4.64507569 |
| *Zm00001d009112* | 6.294750157 | 7.581540925 | 9.373273223 | 4.918936994 |
| *Zm00001d013903* | 6.295605029 | 7.95104545 | 8.199388423 | 4.751626261 |
| *Zm00001d014328* | 6.298500226 | 7.545932483 | 9.000356176 | 4.840849447 |
| *Zm00001d021410* | 6.318168696 | 10.73146427 | 8.187147912 | 5.315569993 |
| *Zm00001d040026* | 6.362123025 | 8.481333867 | 5.148244385 | 4.64507569 |
| *Zm00001d053006* | 6.384989707 | 6.315778261 | 8.324763299 | 4.64507569 |
| *Zm00001d052063* | 6.391199131 | 10.57099936 | 11.13612178 | 5.692171077 |
| *Zm00001d040613* | 6.415827732 | 7.672344661 | 7.69271419 | 4.64507569 |
| *Zm00001d019163* | 6.451068323 | 11.65824624 | 12.54049187 | 6.395236226 |
| *Zm00001d008548* | 6.473571692 | 6.843414897 | 9.375963715 | 4.840849447 |
| *Zm00001d039919* | 6.519464683 | 8.222012143 | 7.11588862 | 4.64507569 |
| *Zm00001d042922* | 6.536505699 | 8.012970317 | 10.34278758 | 5.112423372 |
| *Zm00001d004171* | 6.546460584 | 5.329753003 | 7.701379243 | 4.505258212 |
| *Zm00001d032608* | 6.55713146 | 7.969013943 | 9.572879131 | 4.918936994 |
| *Zm00001d048469* | 6.566828476 | 9.963110031 | 7.739731411 | 4.989073369 |
| *Zm00001d015767* | 6.579069896 | 8.137267718 | 7.42047106 | 4.64507569 |
| *Zm00001d013900* | 6.623601826 | 8.097023779 | 8.600740101 | 4.751626261 |
| *Zm00001d043121* | 6.633113816 | 6.091303685 | 8.623591606 | 4.64507569 |
| *Zm00001d020332* | 6.634246335 | 8.493765838 | 6.742754963 | 4.64507569 |
| *Zm00001d022204* | 6.641994538 | 7.471910876 | 8.184071364 | 4.64507569 |
| *Zm00001d015330* | 6.656285721 | 10.15690701 | 9.098141497 | 5.112423372 |
| *Zm00001d049381* | 6.67614234 | 6.373352119 | 8.616774239 | 4.64507569 |
| *Zm00001d017762* | 6.697833433 | 7.778250684 | 8.051875151 | 4.64507569 |
| *Zm00001d037868* | 6.727761516 | 10.1510455 | 10.53429346 | 5.315569993 |
| *Zm00001d035162* | 6.753777561 | 8.012970317 | 7.928465873 | 4.64507569 |
| *Zm00001d038913* | 6.831517795 | 5.329753003 | 8.901057645 | 4.64507569 |
| *Zm00001d029371* | 7.046198055 | 9.544178439 | 6.888968296 | 4.751626261 |
| *Zm00001d024960* | 7.121548992 | 6.224339817 | 10.43721799 | 4.918936994 |
| *Zm00001d006211* | 7.123774734 | 7.192683251 | 9.569357897 | 4.751626261 |
| *Zm00001d023664* | 7.152242526 | 8.572051238 | 9.695310298 | 4.840849447 |
| *Zm00001d014717* | 7.169879785 | 5.518046476 | 8.25321184 | 4.505258212 |
| *Zm00001d049660* | 7.310705756 | 8.214512044 | 8.674810465 | 4.64507569 |
| *Zm00001d033231* | 7.396499669 | 10.80670675 | 8.264902861 | 4.989073369 |
| *Zm00001d050055* | 7.424054532 | 6.975892528 | 8.156080805 | 4.505258212 |
| *Zm00001d037816* | 7.441251918 | 6.863137744 | 9.337831715 | 4.64507569 |
| *Zm00001d019291* | 7.489200035 | 6.224339817 | 8.452873656 | 4.505258212 |
| *Zm00001d025566* | 7.640183484 | 4.999487261 | 9.693155013 | 4.64507569 |
| *Zm00001d012383* | 7.644256924 | 8.364316324 | 7.141817025 | 4.505258212 |
| *Zm00001d031228* | 7.751859095 | 8.030181053 | 9.39067227 | 4.64507569 |
| *Zm00001d014463* | 7.765435861 | 7.923661392 | 9.446770327 | 4.64507569 |
| *Zm00001d044442* | 7.789119794 | 7.484519917 | 10.25128914 | 4.751626261 |
| *Zm00001d044906* | 7.842599541 | 7.986759142 | 9.521566923 | 4.64507569 |
| *Zm00001d016001* | 7.874619707 | 5.981390978 | 6.425342136 | 4.165048983 |
| *Zm00001d031325* | 7.892161684 | 5.981390978 | 8.899187244 | 4.505258212 |
| *Zm00001d012285* | 7.912637469 | 6.125893033 | 6.358545285 | 4.165048983 |
| *Zm00001d013003* | 7.929820275 | 10.37876485 | 9.442926604 | 4.840849447 |
| *Zm00001d025055* | 7.956334534 | 8.623852678 | 9.389341317 | 4.64507569 |
| *Zm00001d018119* | 7.958655172 | 5.817538239 | 6.607668484 | 4.165048983 |
| *Zm00001d033657* | 8.018489539 | 6.40121846 | 6.226445565 | 4.165048983 |
| *Zm00001d012591* | 8.028533244 | 9.723440613 | 11.2072934 | 4.989073369 |
| *Zm00001d017547* | 8.082450597 | 13.78465737 | 11.45339616 | 5.947996523 |
| *Zm00001d022277* | 8.082552043 | 7.22323831 | 9.972654922 | 4.64507569 |
| *Zm00001d014166* | 8.083140491 | 6.740307105 | 5.828071466 | 4.165048983 |
| *Zm00001d035560* | 8.087634796 | 6.224339817 | 6.478537551 | 4.165048983 |
| *Zm00001d014198* | 8.092074929 | 5.627044899 | 6.817809325 | 4.165048983 |
| *Zm00001d005888* | 8.124691319 | 5.329753003 | 6.941879628 | 4.165048983 |
| *Zm00001d048136* | 8.148099526 | 6.28601 | 6.499233433 | 4.165048983 |
| *Zm00001d039880* | 8.15508702 | 5.258031501 | 6.985674016 | 4.165048983 |
| *Zm00001d020521* | 8.163581223 | 6.532376538 | 6.276051202 | 4.165048983 |
| *Zm00001d050495* | 8.1909842 | 5.677739507 | 6.888968296 | 4.165048983 |
| *Zm00001d049218* | 8.199080805 | 6.28601 | 6.559463526 | 4.165048983 |
| *Zm00001d043789* | 8.199114777 | 6.055734554 | 6.716791337 | 4.165048983 |
| *Zm00001d040826* | 8.21774599 | 5.902250225 | 6.817809325 | 4.165048983 |
| *Zm00001d003188* | 8.250108848 | 4.999487261 | 7.11588862 | 4.165048983 |
| *Zm00001d044079* | 8.269769901 | 4.890152636 | 7.148224597 | 4.165048983 |
| *Zm00001d007341* | 8.281057723 | 11.78221923 | 9.838693666 | 5.053155642 |
| *Zm00001d029154* | 8.296633446 | 6.605302343 | 6.381189668 | 4.165048983 |
| *Zm00001d052209* | 8.379223898 | 4.759138418 | 7.252890259 | 4.165048983 |
| *Zm00001d028873* | 8.388439887 | 5.817538239 | 7.021140947 | 4.165048983 |
| *Zm00001d004524* | 8.406180142 | 5.860646973 | 7.021140947 | 4.165048983 |
| *Zm00001d039245* | 8.435261364 | 5.772790879 | 7.082785391 | 4.165048983 |
| *Zm00001d033447* | 8.447243621 | 5.396499816 | 7.20460858 | 4.165048983 |
| *Zm00001d034723* | 8.458945663 | 6.428507921 | 6.759793413 | 4.165048983 |
| *Zm00001d037730* | 8.460881352 | 7.145562335 | 5.718400596 | 4.165048983 |
| *Zm00001d017381* | 8.471450922 | 4.999487261 | 7.299565859 | 4.165048983 |
| *Zm00001d007395* | 8.478034253 | 6.975892528 | 6.14824761 | 4.165048983 |
| *Zm00001d023941* | 8.478119861 | 5.459064121 | 7.216835796 | 4.165048983 |
| *Zm00001d010075* | 8.496374956 | 6.920665824 | 6.276051202 | 4.165048983 |
| *Zm00001d035559* | 8.519584959 | 9.667041953 | 9.510591968 | 4.64507569 |
| *Zm00001d038490* | 8.523940516 | 5.772790879 | 7.167272907 | 4.165048983 |
| *Zm00001d028750* | 8.539874424 | 10.88882151 | 13.07309699 | 5.402648995 |
| *Zm00001d031257* | 8.54161479 | 5.094861489 | 7.344741326 | 4.165048983 |
| *Zm00001d020100* | 8.555152702 | 6.507158786 | 6.817809325 | 4.165048983 |
| *Zm00001d045862* | 8.582259113 | 5.772790879 | 7.222909625 | 4.165048983 |
| *Zm00001d036370* | 8.58659459 | 7.177151997 | 12.15058796 | 4.989073369 |
| *Zm00001d023387* | 8.595305772 | 4.759138418 | 7.430965151 | 4.165048983 |
| *Zm00001d027740* | 8.597547186 | 6.939320872 | 6.425342136 | 4.165048983 |
| *Zm00001d028778* | 8.613707136 | 6.192360529 | 7.082785391 | 4.165048983 |
| *Zm00001d016128* | 8.620166301 | 4.759138418 | 7.451722212 | 4.165048983 |
| *Zm00001d005609* | 8.633900264 | 6.532376538 | 6.896652419 | 4.165048983 |
| *Zm00001d017486* | 8.639217947 | 6.581430797 | 6.865655363 | 4.165048983 |
| *Zm00001d048335* | 8.657384802 | 5.180251956 | 7.430965151 | 4.165048983 |
| *Zm00001d044777* | 8.662665962 | 4.759138418 | 7.487329087 | 4.165048983 |
| *Zm00001d008528* | 8.67670923 | 6.28601 | 7.102741494 | 4.165048983 |
| *Zm00001d011655* | 8.684158483 | 6.605302343 | 6.904293953 | 4.165048983 |
| *Zm00001d012764* | 8.684258191 | 10.89350925 | 11.16205645 | 4.918936994 |
| *Zm00001d052189* | 8.691037309 | 6.718717969 | 6.817809325 | 4.165048983 |
| *Zm00001d043318* | 8.692888688 | 7.484519917 | 5.148244385 | 4.165048983 |
| *Zm00001d012333* | 8.700563131 | 6.975892528 | 6.549611477 | 4.165048983 |
| *Zm00001d042472* | 8.722014453 | 5.180251956 | 7.487329087 | 4.165048983 |
| *Zm00001d003191* | 8.736349537 | 5.258031501 | 7.487329087 | 4.165048983 |
| *Zm00001d009979* | 8.750456671 | 5.180251956 | 7.51222456 | 4.165048983 |
| *Zm00001d034643* | 8.751176384 | 7.046262483 | 6.53968649 | 4.165048983 |
| *Zm00001d052744* | 8.75533543 | 4.759138418 | 7.565500436 | 4.165048983 |
| *Zm00001d047553* | 8.805468967 | 4.999487261 | 7.58438726 | 4.165048983 |
| *Zm00001d017422* | 8.806666604 | 10.32944815 | 9.182037023 | 4.64507569 |
| *Zm00001d035753* | 8.815749777 | 4.759138418 | 7.616845659 | 4.165048983 |
| *Zm00001d021562* | 8.84766292 | 4.759138418 | 7.644088067 | 4.165048983 |
| *Zm00001d038863* | 8.863986155 | 6.255542868 | 7.322334909 | 4.165048983 |
| *Zm00001d003730* | 8.872888261 | 6.091303685 | 7.399245832 | 4.165048983 |
| *Zm00001d033782* | 8.874010605 | 6.557127854 | 7.173565109 | 4.165048983 |
| *Zm00001d050323* | 8.900091585 | 5.180251956 | 7.644088067 | 4.165048983 |
| *Zm00001d000438* | 8.924481866 | 4.759138418 | 7.709991792 | 4.165048983 |
| *Zm00001d038563* | 8.926366075 | 7.380347725 | 6.312046979 | 4.165048983 |
| *Zm00001d029778* | 8.937649791 | 6.28601 | 7.38851224 | 4.165048983 |
| *Zm00001d019306* | 8.9520388 | 6.344881788 | 7.377696312 | 4.165048983 |
| *Zm00001d010102* | 8.959364549 | 7.208044088 | 6.68138413 | 4.165048983 |
| *Zm00001d020702* | 8.962056794 | 6.581430797 | 7.264705399 | 4.165048983 |
| *Zm00001d046318* | 8.974273085 | 5.180251956 | 7.709991792 | 4.165048983 |
| *Zm00001d004357* | 8.992594501 | 6.428507921 | 7.383114649 | 4.165048983 |
| *Zm00001d042918* | 8.995779461 | 7.080154741 | 6.904293953 | 4.165048983 |
| *Zm00001d038772* | 9.00712515 | 5.942464183 | 7.58438726 | 4.165048983 |
| *Zm00001d022538* | 9.009336495 | 7.380347725 | 6.499233433 | 4.165048983 |
| *Zm00001d029025* | 9.013531914 | 6.125893033 | 10.01035774 | 4.505258212 |
| *Zm00001d014083* | 9.035931933 | 10.11737256 | 10.05635034 | 4.64507569 |
| *Zm00001d002979* | 9.040002449 | 5.860646973 | 7.639583822 | 4.165048983 |
| *Zm00001d040292* | 9.048014133 | 6.532376538 | 7.393889248 | 4.165048983 |
| *Zm00001d006948* | 9.060926405 | 5.627044899 | 7.714278579 | 4.165048983 |
| *Zm00001d047259* | 9.06114297 | 6.957727511 | 7.122416011 | 4.165048983 |
| *Zm00001d044022* | 9.072469177 | 6.803098555 | 7.258810261 | 4.165048983 |
| *Zm00001d025659* | 9.076036433 | 6.019120965 | 7.630532227 | 4.165048983 |
| *Zm00001d021168* | 9.163860187 | 5.677739507 | 7.801440014 | 4.165048983 |
| *Zm00001d019925* | 9.216758119 | 6.224339817 | 7.709991792 | 4.165048983 |
| *Zm00001d037232* | 9.284255139 | 7.977914104 | 12.23353215 | 4.840849447 |
| *Zm00001d019704* | 9.300685863 | 8.294929589 | 11.15659399 | 4.64507569 |
| *Zm00001d044908* | 9.316139769 | 5.772790879 | 7.928465873 | 4.165048983 |
| *Zm00001d024751* | 9.323720833 | 5.459064121 | 7.989739892 | 4.165048983 |
| *Zm00001d047567* | 9.331826396 | 7.353056763 | 7.148224597 | 4.165048983 |
| *Zm00001d027353* | 9.393763409 | 5.942464183 | 7.968412742 | 4.165048983 |
| *Zm00001d030915* | 9.427279292 | 7.471910876 | 7.173565109 | 4.165048983 |
| *Zm00001d037681* | 9.455558032 | 5.518046476 | 8.104926645 | 4.165048983 |
| *Zm00001d008399* | 9.508887593 | 6.782486815 | 7.81346715 | 4.165048983 |
| *Zm00001d048176* | 9.515390195 | 7.932847912 | 6.549611477 | 4.165048983 |
| *Zm00001d028574* | 9.528344453 | 11.0757252 | 9.772871531 | 4.64507569 |
| *Zm00001d006947* | 9.535855142 | 5.258031501 | 8.211525189 | 4.165048983 |
| *Zm00001d006591* | 9.55337546 | 8.088837328 | 6.174849251 | 4.165048983 |
| *Zm00001d027355* | 9.567837459 | 7.569770586 | 7.305292777 | 4.165048983 |
| *Zm00001d028561* | 9.579507584 | 5.942464183 | 8.152936686 | 4.165048983 |
| *Zm00001d052122* | 9.581502247 | 7.253141489 | 7.625984689 | 4.165048983 |
| *Zm00001d051399* | 9.58509249 | 8.121305724 | 6.174849251 | 4.165048983 |
| *Zm00001d033222* | 9.607245915 | 8.088837328 | 6.381189668 | 4.165048983 |
| *Zm00001d043730* | 9.629225623 | 6.091303685 | 8.171698632 | 4.165048983 |
| *Zm00001d009510* | 9.651791767 | 8.08060375 | 6.559463526 | 4.165048983 |
| *Zm00001d016076* | 9.69560133 | 7.683297405 | 7.399245832 | 4.165048983 |
| *Zm00001d043741* | 9.699484799 | 6.740307105 | 8.055249162 | 4.165048983 |
| *Zm00001d051420* | 9.772607817 | 14.30477573 | 12.94531345 | 5.482757748 |
| *Zm00001d037609* | 9.795090168 | 8.436953902 | 5.53096961 | 4.165048983 |
| *Zm00001d028718* | 9.800681257 | 7.847958473 | 11.73703742 | 4.64507569 |
| *Zm00001d032519* | 9.877671297 | 6.782486815 | 8.244380543 | 4.165048983 |
| *Zm00001d007411* | 9.885837137 | 7.238269593 | 8.051875151 | 4.165048983 |
| *Zm00001d009626* | 9.89097132 | 7.238269593 | 8.05861523 | 4.165048983 |
| *Zm00001d010586* | 9.953555881 | 10.53844422 | 11.32083627 | 4.64507569 |
| *Zm00001d025229* | 10.00774774 | 6.315778261 | 8.512880213 | 4.165048983 |
| *Zm00001d050577* | 10.09761435 | 7.521686288 | 8.171698632 | 4.165048983 |
| *Zm00001d046824* | 10.10012961 | 8.176410786 | 7.531830309 | 4.165048983 |
| *Zm00001d028999* | 10.11538689 | 6.344881788 | 8.619050291 | 4.165048983 |
| *Zm00001d025753* | 10.12848326 | 6.159560997 | 8.666032963 | 4.165048983 |
| *Zm00001d011755* | 10.20123473 | 5.981390978 | 8.765930808 | 4.165048983 |
| *Zm00001d039094* | 10.26062125 | 5.860646973 | 8.839964575 | 4.165048983 |
| *Zm00001d048348* | 10.46521311 | 8.542444377 | 7.772975247 | 4.165048983 |
| *Zm00001d010010* | 10.4708594 | 8.583725181 | 7.714278579 | 4.165048983 |
| *Zm00001d003301* | 10.55013841 | 7.977914104 | 8.529919867 | 4.165048983 |
| *Zm00001d019504* | 10.68260847 | 8.595305012 | 8.177898364 | 4.165048983 |
| *Zm00001d023700* | 10.73157223 | 8.937502325 | 7.69705332 | 4.165048983 |
| *Zm00001d016152* | 10.80540326 | 8.589526765 | 8.429708234 | 4.165048983 |
| *Zm00001d044124* | 11.35383535 | 8.287802566 | 9.494590208 | 4.165048983 |
| *Zm00001d037894* | 11.39125756 | 12.1332779 | 13.51785703 | 4.751626261 |
| *Zm00001d043912* | 11.44785661 | 7.55790167 | 9.828911764 | 4.165048983 |
| *Zm00001d010828* | 11.57002143 | 7.208044088 | 10.02328131 | 4.165048983 |
| *Zm00001d043709* | 11.87049166 | 9.614040384 | 9.384005174 | 4.165048983 |
| *Zm00001d027832* | 12.72527759 | 7.788420268 | 11.19478462 | 4.165048983 |
| *Zm00001d019629* | 12.76814356 | 7.177151997 | 11.28588021 | 4.165048983 |
| *Zm00001d045321* | 13.06620716 | 10.92246742 | 10.33727902 | 4.165048983 |
| *Zm00001d035178* | 22.21909875 | 4.165048983 | 8.450318109 | 4.165048983 |
| *Zm00001d021666* | 23.68752487 | 4.165048983 | 9.909087728 | 4.165048983 |

1. *Differentially expressed genes in leaves of maize (down-regulated) in response to combined salinity and boron stress*

| **Gene ID** | **Log2FC** | **3 h** | **96 h** | **0 h** |
| --- | --- | --- | --- | --- |
| *Zm00001d034017* | -2.64200698 | 8.449774544 | 8.014225668 | 10.743484 |
| *Zm00001d028742* | -2.710949045 | 8.843598315 | 8.452873656 | 11.261558 |
| *Zm00001d031659* | -2.813117502 | 9.038420177 | 8.838012969 | 11.663049 |
| *Zm00001d020414* | -2.816956717 | 7.080154741 | 7.210735527 | 9.6405311 |
| *Zm00001d017069* | -2.951770942 | 6.581430797 | 6.499233433 | 8.9780679 |
| *Zm00001d042812* | -2.975843336 | 6.76155977 | 6.911893389 | 9.402625 |
| *Zm00001d029768* | -2.984128576 | 6.651814267 | 6.607668484 | 9.1304758 |
| *Zm00001d044194* | -3.009385746 | 7.096796458 | 6.963950001 | 9.6780985 |
| *Zm00001d044017* | -3.016968736 | 9.561963995 | 8.276499267 | 11.986794 |
| *Zm00001d039120* | -3.047120932 | 7.339210402 | 7.014119711 | 9.9047545 |
| *Zm00001d023314* | -3.08918386 | 7.325226493 | 6.66332816 | 9.7504176 |
| *Zm00001d020557* | -3.13796991 | 7.208044088 | 6.978470465 | 9.8834737 |
| *Zm00001d047307* | -3.350442324 | 7.59321434 | 6.064911484 | 9.9939781 |
| *Zm00001d041007* | -3.361739748 | 6.344881788 | 5.699063799 | 8.6361542 |
| *Zm00001d036175* | -3.397061325 | 6.975892528 | 6.626477589 | 9.7621257 |
| *Zm00001d044970* | -3.410047477 | 8.923801553 | 6.742754963 | 11.458353 |
| *Zm00001d024291* | -3.440467977 | 6.993822424 | 5.944347934 | 9.4633244 |
| *Zm00001d029075* | -3.445941996 | 7.063311783 | 6.403450688 | 9.752096 |
| *Zm00001d024409* | -3.580066292 | 7.60479244 | 7.603025775 | 10.931435 |
| *Zm00001d010634* | -3.644064751 | 5.860646973 | 5.384877491 | 8.1715365 |
| *Zm00001d028770* | -3.808492442 | 6.159560997 | 5.618218608 | 8.807585 |
| *Zm00001d016274* | -3.812519356 | 9.254431335 | 6.941879628 | 12.19663 |
| *Zm00001d023869* | -3.831936807 | 6.373352119 | 5.21337153 | 8.8363835 |
| *Zm00001d029031* | -3.904957801 | 6.455245719 | 5.21337153 | 9.0036767 |
| *Zm00001d044018* | -3.906106339 | 7.433394342 | 6.25148661 | 10.448791 |
| *Zm00001d047096* | -3.938252235 | 5.817538239 | 5.618218608 | 8.5880177 |
| *Zm00001d052532* | -4.064016367 | 6.740307105 | 5.21337153 | 9.4977531 |
| *Zm00001d038718* | -4.095408741 | 6.843414897 | 5.113735695 | 9.6222918 |
| *Zm00001d038250* | -4.128632565 | 5.459064121 | 4.91429289 | 7.6772419 |
| *Zm00001d011890* | -4.13519643 | 5.180251956 | 5.331069118 | 7.8258157 |
| *Zm00001d029814* | -4.196705471 | 5.902250225 | 5.077732888 | 8.4906519 |
| *Zm00001d023781* | -4.274159063 | 6.224339817 | 4.697786404 | 8.852137 |
| *Zm00001d037694* | -4.35225698 | 4.759138418 | 5.113735695 | 7.2577542 |
| *Zm00001d037513* | -4.354585665 | 8.858080682 | 5.845391089 | 12.192928 |
| *Zm00001d050416* | -4.412707826 | 4.759138418 | 5.077732888 | 7.2384633 |
| *Zm00001d040265* | -4.48622106 | 5.726251078 | 4.62706207 | 8.22587 |
| *Zm00001d033846* | -4.637639763 | 5.627044899 | 4.866864674 | 8.3332243 |
| *Zm00001d008957* | -4.689779227 | 5.459064121 | 4.697786404 | 7.9661714 |
| *Zm00001d009425* | -4.786473961 | 4.890152636 | 4.815701458 | 7.1990665 |
| *Zm00001d041148* | -4.911908567 | 5.094861489 | 5.331069118 | 8.4288965 |
| *Zm00001d012738* | -5.044184805 | 4.999487261 | 4.759836024 | 7.4870796 |
| *Zm00001d024784* | -5.044748089 | 5.180251956 | 4.542814985 | 7.5739085 |
| *Zm00001d038126* | -5.102973315 | 4.165048983 | 5.274078324 | 7.9427324 |
| *Zm00001d045302* | -5.173761694 | 5.180251956 | 5.000454077 | 8.2594026 |
| *Zm00001d017429* | -5.329582037 | 4.165048983 | 5.274078324 | 8.1461409 |
| *Zm00001d011548* | -5.693841754 | 4.165048983 | 4.697786404 | 6.7830188 |
| *Zm00001d039833* | -6.263650109 | 4.759138418 | 4.165048983 | 6.9599416 |
| *Zm00001d035767* | -6.504590138 | 4.999487261 | 4.542814985 | 8.3511006 |
| *Zm00001d038505* | -6.978678033 | 4.586606736 | 4.62706207 | 7.9892318 |
| *Zm00001d012259* | -7.633706634 | 4.165048983 | 4.542814985 | 7.611683 |
| *Zm00001d029992* | -8.152121352 | 4.165048983 | 4.165048983 | 6.3771165 |
| *Zm00001d011768* | -8.524094394 | 4.165048983 | 4.165048983 | 6.6259443 |
| *Zm00001d026574* | -9.003266232 | 4.165048983 | 4.165048983 | 6.9717514 |
| *Zm00001d013766* | -12.61925983 | 4.165048983 | 4.165048983 | 10.187472 |

1. *List of Differentially expressed genes in roots of maize (up-regulated) in response to combined salinity and boron stress*

| **Gene ID** | **Log2FC** | **3 h** | **96 h** | **0 h** |
| --- | --- | --- | --- | --- |
| *Zm00001d052253* | 3.07723449 | 9.32961309 | 9.51122878 | 6.94923816 |
| *Zm00001d034940* | 3.093485774 | 10.6990834 | 11.8107061 | 8.46721857 |
| *Zm00001d053212* | 3.110267096 | 9.95390345 | 9.53222681 | 7.16506533 |
| *Zm00001d029151* | 3.129504573 | 9.9944684 | 9.56752548 | 7.17974361 |
| *Zm00001d028303* | 3.200244279 | 9.42738919 | 10.5764745 | 7.3637132 |
| *Zm00001d024027* | 3.222336715 | 10.5867192 | 11.1223329 | 7.95175752 |
| *Zm00001d021647* | 3.228248854 | 10.171835 | 11.4033038 | 7.9765788 |
| *Zm00001d029087* | 3.241101751 | 9.46398854 | 10.5663415 | 7.3379819 |
| *Zm00001d002266* | 3.305411861 | 12.2675192 | 10.6794522 | 8.56802314 |
| *Zm00001d015658* | 3.337629516 | 10.8475657 | 11.848817 | 8.32387421 |
| *Zm00001d038806* | 3.363707215 | 9.01357272 | 9.02722268 | 6.51991608 |
| *Zm00001d033273* | 3.39384373 | 9.33949839 | 10.3163448 | 7.08132522 |
| *Zm00001d024702* | 3.427279729 | 9.34146731 | 10.5936813 | 7.19426331 |
| *Zm00001d016255* | 3.429709079 | 9.56682943 | 8.33294242 | 6.51991608 |
| *Zm00001d017240* | 3.571089079 | 10.6975492 | 9.00590553 | 7.08915885 |
| *Zm00001d012420* | 3.57517117 | 10.7737797 | 10.8914033 | 7.63989157 |
| *Zm00001d033718* | 3.588861687 | 9.70773687 | 8.61866817 | 6.54408 |
| *Zm00001d038181* | 3.676967425 | 8.03321917 | 8.00993019 | 5.85891945 |
| *Zm00001d025823* | 3.708214381 | 8.13745931 | 8.41317482 | 5.96557135 |
| *Zm00001d028230* | 3.709706411 | 11.7188478 | 10.7918053 | 7.93073356 |
| *Zm00001d032295* | 3.757927478 | 10.0484434 | 8.18061986 | 6.53205828 |
| *Zm00001d036402* | 3.790954163 | 8.08155576 | 7.8057444 | 5.78848897 |
| *Zm00001d037868* | 3.822117793 | 8.59442009 | 8.00993019 | 5.94517099 |
| *Zm00001d008795* | 3.830080733 | 8.14201977 | 8.04407133 | 5.83604896 |
| *Zm00001d018298* | 3.865357375 | 9.16811063 | 8.29106285 | 6.14964894 |
| *Zm00001d020702* | 3.866280438 | 8.58777871 | 9.03986258 | 6.16639116 |
| *Zm00001d045361* | 3.998020334 | 9.53794354 | 8.65204942 | 6.27690547 |
| *Zm00001d026334* | 4.022128464 | 10.7918644 | 10.0639638 | 7.05754343 |
| *Zm00001d014325* | 4.076938177 | 8.69976814 | 9.03986258 | 6.09778353 |
| *Zm00001d025665* | 4.126090333 | 7.84404457 | 9.14887553 | 5.96557135 |
| *Zm00001d028815* | 4.147732324 | 10.3860783 | 12.4300813 | 7.91796673 |
| *Zm00001d048898* | 4.157145392 | 8.2557162 | 10.8310235 | 6.70108956 |
| *Zm00001d033987* | 4.22210803 | 8.75707885 | 7.17058962 | 5.73819758 |
| *Zm00001d024778* | 4.230378778 | 12.4021417 | 10.0619039 | 7.78817425 |
| *Zm00001d034978* | 4.29607692 | 9.28733609 | 8.53163398 | 6.04321272 |
| *Zm00001d036513* | 4.316866547 | 9.24588209 | 8.1652224 | 5.96557135 |
| *Zm00001d049244* | 4.320592707 | 8.6368524 | 6.9836933 | 5.65659696 |
| *Zm00001d044078* | 4.323565678 | 10.5214474 | 8.50749987 | 6.48273794 |
| *Zm00001d053234* | 4.340760852 | 7.32517181 | 8.54354967 | 5.65659696 |
| *Zm00001d028630* | 4.341016332 | 11.8911078 | 10.9359283 | 7.57037917 |
| *Zm00001d019726* | 4.350341284 | 7.06421448 | 7.62466817 | 5.4224417 |
| *Zm00001d004921* | 4.366519477 | 8.7302176 | 9.39811379 | 6.07991095 |
| *Zm00001d044156* | 4.372682885 | 7.21439392 | 7.54266529 | 5.4224417 |
| *Zm00001d028814* | 4.380789376 | 10.9210494 | 13.2993094 | 8.40191309 |
| *Zm00001d012527* | 4.404580885 | 8.29304202 | 8.81800901 | 5.83604896 |
| *Zm00001d028744* | 4.419294571 | 10.2196613 | 8.10190183 | 6.26177704 |
| *Zm00001d021775* | 4.419863233 | 10.4951948 | 9.77297703 | 6.62507416 |
| *Zm00001d005813* | 4.427778544 | 8.08629971 | 7.05473555 | 5.49780954 |
| *Zm00001d028718* | 4.427819519 | 9.13895843 | 9.45263907 | 6.14964894 |
| *Zm00001d047077* | 4.479525497 | 8.55410046 | 7.64723386 | 5.65659696 |
| *Zm00001d021726* | 4.51216559 | 7.23203799 | 8.10190183 | 5.49780954 |
| *Zm00001d027901* | 4.566913138 | 9.17916731 | 6.47520376 | 5.71188929 |
| *Zm00001d028999* | 4.570364311 | 7.09368908 | 7.36240326 | 5.33636539 |
| *Zm00001d007901* | 4.609901123 | 11.5399783 | 8.08561885 | 6.81198039 |
| *Zm00001d046134* | 4.627802034 | 6.99277857 | 8.96671773 | 5.65659696 |
| *Zm00001d023664* | 4.697898294 | 9.7950314 | 9.07712771 | 6.11535609 |
| *Zm00001d042880* | 4.718479626 | 8.78634508 | 8.53760426 | 5.76371057 |
| *Zm00001d023596* | 4.736398504 | 6.93927103 | 7.66943907 | 5.33636539 |
| *Zm00001d037005* | 4.753062853 | 6.88347801 | 9.21720855 | 5.68471597 |
| *Zm00001d023294* | 4.778191682 | 7.71448444 | 8.32604761 | 5.53249337 |
| *Zm00001d048947* | 4.785247511 | 6.68670452 | 10.4148389 | 6.09778353 |
| *Zm00001d033300* | 4.85633617 | 9.60828232 | 7.51831468 | 5.81258608 |
| *Zm00001d028574* | 4.872262723 | 9.87462073 | 6.84927463 | 5.85891945 |
| *Zm00001d013099* | 4.87405538 | 7.96264008 | 6.60463323 | 5.33636539 |
| *Zm00001d017381* | 4.900341301 | 6.78888011 | 8.36019225 | 5.4224417 |
| *Zm00001d019078* | 4.928776075 | 11.7649782 | 9.40463687 | 6.84062238 |
| *Zm00001d010131* | 4.94534814 | 8.46987431 | 6.52864426 | 5.4224417 |
| *Zm00001d038870* | 5.017946853 | 6.83704813 | 7.38965067 | 5.2336211 |
| *Zm00001d024210* | 5.018279617 | 6.33449664 | 11.3118417 | 6.40471194 |
| *Zm00001d028307* | 5.021276718 | 7.90996983 | 7.84514503 | 5.4224417 |
| *Zm00001d044442* | 5.068022493 | 11.1575477 | 11.3282077 | 6.89606568 |
| *Zm00001d046981* | 5.071177716 | 6.53098533 | 7.61324633 | 5.2336211 |
| *Zm00001d046644* | 5.10245058 | 8.34922331 | 6.82888198 | 5.3810132 |
| *Zm00001d033797* | 5.182429013 | 8.94002538 | 8.3802925 | 5.62743798 |
| *Zm00001d011315* | 5.194536544 | 6.92830198 | 9.46517362 | 5.62743798 |
| *Zm00001d050805* | 5.23158295 | 6.26084061 | 9.24656575 | 5.53249337 |
| *Zm00001d014121* | 5.245822166 | 6.18198325 | 9.79050479 | 5.68471597 |
| *Zm00001d047399* | 5.251518678 | 6.78888011 | 8.01854322 | 5.28766199 |
| *Zm00001d050055* | 5.285383727 | 7.98316862 | 8.65753795 | 5.49780954 |
| *Zm00001d012591* | 5.295783268 | 8.8761695 | 7.93906331 | 5.53249337 |
| *Zm00001d038221* | 5.323953688 | 9.42738919 | 7.23255609 | 5.59712775 |
| *Zm00001d010586* | 5.330453969 | 9.27708416 | 8.62988211 | 5.68471597 |
| *Zm00001d019411* | 5.350337537 | 6.26084061 | 7.97494222 | 5.2336211 |
| *Zm00001d044147* | 5.369950256 | 6.14030737 | 8.95341352 | 5.4224417 |
| *Zm00001d009084* | 5.371725573 | 7.83840844 | 7.8156989 | 5.33636539 |
| *Zm00001d022032* | 5.382838898 | 10.2143057 | 6.52864426 | 5.78848897 |
| *Zm00001d008983* | 5.464526377 | 7.61207774 | 8.1418078 | 5.33636539 |
| *Zm00001d037894* | 5.47415409 | 10.8413275 | 9.50213495 | 6.16639116 |
| *Zm00001d044022* | 5.483461762 | 7.22324479 | 7.12216353 | 5.17208745 |
| *Zm00001d025753* | 5.495839784 | 6.29826735 | 7.71281444 | 5.17208745 |
| *Zm00001d010828* | 5.511666989 | 7.3734056 | 8.01854322 | 5.28766199 |
| *Zm00001d028816* | 5.517798068 | 8.7059101 | 9.22091118 | 5.62743798 |
| *Zm00001d027443* | 5.531542289 | 8.57103888 | 6.80816056 | 5.33636539 |
| *Zm00001d010218* | 5.535896672 | 5.95465812 | 9.12930255 | 5.4224417 |
| *Zm00001d018064* | 5.574850889 | 8.90845299 | 11.8168231 | 6.45729558 |
| *Zm00001d047993* | 5.606865825 | 9.55670049 | 6.72173709 | 5.53249337 |
| *Zm00001d042811* | 5.608496287 | 7.25807939 | 7.26250379 | 5.17208745 |
| *Zm00001d031878* | 5.625776719 | 8.01838957 | 9.42081628 | 5.56553326 |
| *Zm00001d002847* | 5.64159283 | 9.08588544 | 8.67928373 | 5.56553326 |
| *Zm00001d043741* | 5.702534928 | 8.35707106 | 5.92195101 | 5.2336211 |
| *Zm00001d012510* | 5.751848213 | 7.86081836 | 7.44257063 | 5.2336211 |
| *Zm00001d052243* | 5.773871869 | 6.36961571 | 10.2954613 | 5.68471597 |
| *Zm00001d035753* | 5.784985242 | 6.80109296 | 9.26461399 | 5.4224417 |
| *Zm00001d032138* | 5.81588184 | 6.64614301 | 7.29180352 | 5.09886375 |
| *Zm00001d042114* | 5.833122482 | 10.2824221 | 8.28396135 | 5.73819758 |
| *Zm00001d012456* | 5.839728505 | 10.1685156 | 6.36051815 | 5.62743798 |
| *Zm00001d003301* | 5.884125171 | 7.74500191 | 7.79571863 | 5.2336211 |
| *Zm00001d010899* | 5.90386988 | 8.44801693 | 6.4475613 | 5.2336211 |
| *Zm00001d031811* | 5.924271255 | 6.64614301 | 9.7501232 | 5.49780954 |
| *Zm00001d017547* | 5.954509965 | 12.9374058 | 8.38693 | 6.82159886 |
| *Zm00001d021562* | 6.100276273 | 6.05168885 | 8.71129468 | 5.2336211 |
| *Zm00001d000438* | 6.100276273 | 6.05168885 | 8.71129468 | 5.2336211 |
| *Zm00001d029025* | 6.110142766 | 6.00434979 | 9.27533512 | 5.33636539 |
| *Zm00001d053746* | 6.174369416 | 9.33159559 | 6.00904881 | 5.33636539 |
| *Zm00001d014083* | 6.183463544 | 7.65136903 | 6.67620499 | 5.09886375 |
| *Zm00001d038913* | 6.214279812 | 8.75707885 | 10.6134998 | 5.73819758 |
| *Zm00001d031228* | 6.217696099 | 7.78076398 | 6.4475613 | 5.09886375 |
| *Zm00001d027619* | 6.268021095 | 11.809679 | 8.78832224 | 6.11535609 |
| *Zm00001d047582* | 6.32318221 | 10.0532514 | 8.17294204 | 5.53249337 |
| *Zm00001d045404* | 6.356095923 | 7.97806445 | 6.23316248 | 5.09886375 |
| *Zm00001d021727* | 6.386932494 | 6.99277857 | 7.71281444 | 5.09886375 |
| *Zm00001d025964* | 6.447226064 | 7.94180792 | 6.62895218 | 5.09886375 |
| *Zm00001d027948* | 6.54675782 | 7.49429119 | 9.19479015 | 5.28766199 |
| *Zm00001d019704* | 6.565192538 | 8.21305865 | 9.26102243 | 5.33636539 |
| *Zm00001d043180* | 6.582249756 | 8.31336257 | 5.35787606 | 5.09886375 |
| *Zm00001d003190* | 6.643394826 | 7.59872956 | 11.5624893 | 5.83604896 |
| *Zm00001d007341* | 6.647483768 | 10.3464852 | 8.50749987 | 5.53249337 |
| *Zm00001d048307* | 6.647545535 | 8.32141014 | 5.82609586 | 5.09886375 |
| *Zm00001d027949* | 6.683077071 | 8.7511531 | 7.99254473 | 5.2336211 |
| *Zm00001d042922* | 6.684682926 | 10.1097298 | 9.23562697 | 5.53249337 |
| *Zm00001d044050* | 6.73987098 | 5.65601041 | 9.34143924 | 5.2336211 |
| *Zm00001d031877* | 6.885130537 | 5.95465812 | 10.2364206 | 5.3810132 |
| *Zm00001d006526* | 7.054636136 | 7.00322339 | 8.45165706 | 5.09886375 |
| *Zm00001d006948* | 7.145959319 | 8.80362189 | 5.44712095 | 5.09886375 |
| *Zm00001d024522* | 7.28801114 | 9.86097204 | 5.52522723 | 5.2336211 |
| *Zm00001d025055* | 7.295746165 | 8.28483174 | 7.88346899 | 5.09886375 |
| *Zm00001d002799* | 7.334499495 | 7.95226253 | 8.29106285 | 5.09886375 |
| *Zm00001d053195* | 7.33906482 | 8.96069389 | 5.71875888 | 5.09886375 |
| *Zm00001d027947* | 7.461653494 | 8.31739205 | 8.14965576 | 5.09886375 |
| *Zm00001d036164* | 7.518001549 | 6.18198325 | 9.08933746 | 5.09886375 |
| *Zm00001d045321* | 7.6602178 | 8.19562991 | 6.50222084 | 5.00312751 |
| *Zm00001d017422* | 7.70096245 | 10.061627 | 7.73400632 | 5.2336211 |
| *Zm00001d025082* | 7.819781607 | 5.40166472 | 9.42723742 | 5.09886375 |
| *Zm00001d044906* | 7.849306419 | 10.2803735 | 7.36240326 | 5.2336211 |
| *Zm00001d006591* | 8.102881469 | 9.64698436 | 6.19892273 | 5.09886375 |
| *Zm00001d043121* | 8.658809567 | 5.95465812 | 10.7743062 | 5.17208745 |
| *Zm00001d006211* | 8.730924239 | 6.68670452 | 6.67620499 | 4.77115123 |
| *Zm00001d027832* | 8.763081188 | 6.36961571 | 6.96531751 | 4.77115123 |
| *Zm00001d029366* | 8.832485605 | 7.11298242 | 6.19892273 | 4.77115123 |
| *Zm00001d053225* | 8.840102247 | 7.10337065 | 6.23316248 | 4.77115123 |
| *Zm00001d024665* | 8.886030582 | 6.05168885 | 7.23255609 | 4.77115123 |
| *Zm00001d046824* | 8.942262648 | 7.34144004 | 5.82609586 | 4.77115123 |
| *Zm00001d012285* | 9.024133977 | 7.38128144 | 5.92195101 | 4.77115123 |
| *Zm00001d023700* | 9.102646779 | 7.47242066 | 5.82609586 | 4.77115123 |
| *Zm00001d011299* | 9.11895394 | 5.78782146 | 7.50597637 | 4.77115123 |
| *Zm00001d051149* | 9.293730802 | 7.58525163 | 6.00904881 | 4.77115123 |
| *Zm00001d016151* | 9.295331802 | 7.72064156 | 5.25129923 | 4.77115123 |
| *Zm00001d007411* | 9.396267191 | 7.21439392 | 6.96531751 | 4.77115123 |
| *Zm00001d052371* | 9.643712581 | 7.17839721 | 7.34857297 | 4.77115123 |
| *Zm00001d002564* | 9.710940528 | 7.55097157 | 7.0196922 | 4.77115123 |
| *Zm00001d025229* | 9.830064369 | 7.45764481 | 7.33460099 | 4.77115123 |
| *Zm00001d019504* | 9.884052813 | 8.05759419 | 6.23316248 | 4.77115123 |
| *Zm00001d014253* | 9.945021459 | 8.23027585 | 5.52522723 | 4.77115123 |
| *Zm00001d044529* | 10.09203802 | 8.14656558 | 6.62895218 | 4.77115123 |
| *Zm00001d013903* | 10.22237322 | 8.18241631 | 6.90858968 | 4.77115123 |
| *Zm00001d013900* | 10.36531294 | 8.30120514 | 7.00181571 | 4.77115123 |
| *Zm00001d032999* | 10.37810277 | 7.11298242 | 8.27682418 | 4.77115123 |
| *Zm00001d033050* | 10.42651344 | 8.56766723 | 6.23316248 | 4.77115123 |
| *Zm00001d027900* | 10.59955871 | 8.08155576 | 7.88346899 | 4.77115123 |
| *Zm00001d044762* | 22.96073699 | 8.76885724 | 4.77115123 | 4.77115123 |
| *Zm00001d025141* | 23.55104695 | 9.32961309 | 4.77115123 | 4.77115123 |

**2. Supplementary table S2:** Functions of the commonly upregulated DEGs in leaf and root derived from UniProt.

| **Functional group** | **Gene ID** | **UniProt ID** | **Protein name** | **Length (aa)** | **Function** | **Reference** |
| --- | --- | --- | --- | --- | --- | --- |
| **I**  **D E F E N S E** | *Zm00001d010586* | B6UH30 | PEBP (Phosphatidylethanolamine-binding protein) family protein | 174 | Biosynthesis of polysaccharides and glycoproteins in the plant cell wall | [1] |
|  | *Zm00001d019504* | A0A1D6HXY5 | Plasma membrane associated protein | 33 | Plasma membrane related | [2] |
|  | *Zm00001d045321* | B4FKQ0 | Dehydrin family protein expressed | 326 | Protects against dehydration | [3] |
|  | *Zm00001d028718* | B6SPH7 | Hydrophobic protein RCI2B | 54 | Regulation of membrane potential | [4] |
|  | *Zm00001d025753* | C0P451 | Chitinase (EC 3.2.1.14) | 281 | Chitin production | [5] |
|  | *Zm00001d008795* | K7UQW6 | Glycosyltransferase (EC 2.4.1.-) | 476 | Polysaccharide biosynthesis in cell wall | [1] |
|  | *Zm00001d038913* | B6U7P4 | Lipid transfer protein1 (Nonspecific lipid-transfer protein AKCS9) | 98 | Transfer of phospholipids, reproductive development, pathogen defense and abiotic stress response | [6] |
|  | *Zm00001d006948* | B8A062 | Cytochrome P450 709B2 | 529 | Biosynthesis of lignins, terpenes, alkaloids and other secondary compounds for plant defense | [7] |
|  | *Zm00001d038221* | B4FJN5 | NAC domain containing protein 32 (NAC protein) (NAC transcription factor) | 339 | Plant defense related TF | [8] |
|  | *Zm00001d044906* | A0A1D6NS66 | 12-oxo-phytodienoic acid reductase2 | 121 | Wound induced gene expression | [9] |
|  | *Zm00001d002847* | C4JBV8 | (+)-neomenthol dehydrogenase | 314 | Resistance against microbial pathogens | [10] |
|  | *Zm00001d047399* | K7VLG8 | Glucan endo-1,3-beta-D-glucosidase (EC 3.2.1.39) | 451 | Important component of cell walls in the Poaceae family of higher plants | [11] |
|  | *Zm00001d052253* | C0HHL6 | F-box/kelch-repeat protein SKIP11 | 423 | Production of green leaf volatiles for plant defense during stress/pathogen attack | [12] |
|  | *Zm00001d045361* | B6U3H7 | Uncharacterized protein | 121 | Integral Component of membrane | [13] |
|  | *Zm00001d035753* | A0A1D6LII8 | Polygalacturonase inhibitor | 341 | Inhibits endopolygalacturonases secreted by pathogens | [14] |
|  | *Zm00001d029151* | A0A1D6K338 | UDP-glucose 4-epimerase 4 | 82 | Regulation of carbohydrate biosynthesis of cell wall | [15] |
|  | *Zm00001d037005* | A0A1D6LT92 | Putative FAD-binding Berberine family protein | 524 | Metabolism of cell wall related defense signal compounds like cellodextrose and cellodextrin | [16] |
|  | *Zm00001d025229* | B6SI10 | Uncharacterized protein | 275 | Integral Component of membrane | [17] |
|  | *Zm00001d003190* | B4FTS6 | Chitinase (EC 3.2.1.14) | 280 | Confer pathogen/disease resistance through chitin production | [18] |
|  | *Zm00001d028230* | A0A1D6JTD3 | Sugar transport protein 13 | 514 | Triggers plant defense reaction by changing sugar fluxes towards host cell | [19] |
| **II**  **REGULATION** | *Zm00001d007411* | A0A1D6F696 | NADH-ubiquinone reductase complex 1 MLRQ subunit | 175 | ATP Generation | [20] |
|  | *Zm00001d044022* | A0A1D6NH20 | Seed maturation protein PM41 | 85 | Protects biomolecules from inactivation by conformational changes during seed aging | [21] |
|  | *Zm00001d053212* | A0A1D6QMT0 | E3 ubiquitin ligase BIG BROTHER-related | 273 | Protein targeting for ubiquitylation | [22] |
|  | *Zm00001d014083* | A0A1D6GPU1 | Beta-amylase | 62 | Starch degradation, crucial for DNA Sequence Recognition and Transcriptional Activation | [23] |
|  | *Zm00001d042880* | A0A1D6N781 | Regulator of chromosome condensation1 | 506 | Seed Development | [24] |
|  | *Zm00001d024522* | K7UFH5 | Putative HLH DNA-binding domain superfamily protein | 258 | Transcriptional regulators | [25] |
|  | *Zm00001d002799* | A0A060CYX6 | HB transcription factor (Homeobox-leucine zipper protein ATHB-12) (Fragment) | 261 | Ion homeostasis in plants | [26] |
|  | *Zm00001d024778* | C0HIJ6 | Hydrophobic protein RCI2B | 58 | Regulation of membrane potential | [4] |
|  | *Zm00001d006211* | B4FTC3 | Protein STAY-GREEN 1 chloroplastic (Senescence-inducible chloroplast stay-green protein 1) | 284 | Chlorophyll degradation during leaf senescence and fruit ripening | [27] |
|  | *Zm00001d042922* | A0A1D6N7I2 | Putative mediator of RNA polymerase II transcription subunit 37c | 234 | Control of transcript elongation | [28] |
|  | *Zm00001d018298* | B4F9E8 | 17.4 kDa class III heat shock protein | 171 | Prevention of protein denaturation during stress as ATP-independent chaperon | [29] |
|  | *Zm00001d018064* | A0A1D6HKK7 | Laccase-14 | 188 | Reduce oxygen to water without producing harmful by-products | [30] |
|  | *Zm00001d044442* | A0A1D6NLT5 | ABC transporter G family member 40 | 1455 | Transporters involved in detoxification process, organ growth, plant nutrition, plant development, response to abiotic stresses, pathogen resistance | [46] |
|  | *Zm00001d033273* | A5YMH6 | Major facilitator superfamily protein (Organic cation/carnitine transporter 7) (Synaptic vesicle 2-related protein) | 479 | Gene regulation for transporters in tonoplast for stress adaptation | [32] |
|  | *Zm00001d043121* | A0A1D6N932 | Osmotin-like protein OSM34 | 220 | Enhanced tolerance against drought, salinity, oxidative stress, and the charcoal rot pathogen | [33] |
|  | *Zm00001d007901* | A0A1D6F9P3 | Cylicin-1 | 224 | Drought tolerance | [34] |
|  | *Zm00001d017547* | A0A1D6HFK1 | Dehydrin COR410 | 61 | Osmotic stress tolerance | [35] |
|  | *Zm00001d025665* | A0A1D6J8G2 | Putative amino acid permease 7 | 735 | Root import, long distance translocation and stress response | [36] |
|  | *Zm00001d038806* | A0A1D6MAR6 | Chaperone protein ClpB1 | 277 | Help in protein aggregation during stress | [37] |
|  | *Zm00001d012285* | A0A1D6G7Z2 | Putative MYB DNA-binding domain superfamily protein | 103 | Regulation of biotic and abiotic stress responses, metabolism and defense | [38] |
|  | *Zm00001d032295* | A0A1D6KPR1 | DRE-binding protein 1 | 262 | Transcriptional repression of DRE mediated gene for abiotic stress tolerance | [39] |
|  | *Zm00001d029087* | A0A1D6K2D6 | Sucrose synthase (EC 2.4.1.13) | 87 | Energy production. plant-metabolite production and synthesis of complex carbohydrates | [40] |
|  | *Zm00001d027832* | A0A1D6JQ00 | Embryonic protein DC-8 | 352 | Regulation of embryo maturation and desiccation | [41] |
|  | *Zm00001d029025* | A0A1D6K1T6 | Phosphoglycerate mutase (2,3-diphosphoglycerate-independent) (EC 5.4.2.12) | 565 | Involved in stomatal movement, vegetative growth and pollen production | [42] |
|  | *Zm00001d027619* | A0A1D6JNC2 | Beta amylase2 | 69 | Breakdown of starch to maltose | [43] |
|  | *Zm00001d012420* | A0A1D6G8N2 | Heat shock 70 kDa protein | 234 | Cellular homeostasis | [44] |
|  | *Zm00001d006591* | A0A1D6EY40 | F-box domain containing protein | 170 | Regulation plant development and growth | [45] |
|  | *Zm00001d023294* | C4JB32 | NAC transcription factor 29 | 475 | Important for senescence and response to salt and drought stress | [46] |
| **III**  **SIGNALLING** | *Zm00001d014325* | A0A1D6GS24 | Calcium-binding EF-hand family protein | 157 | Ca2+ regulation in plants | [47] |
|  | *Zm00001d029366* | A0A1D6K4N2 | Transducin/WD40 repeat-like superfamily protein | 453 | Involved in signal transduction, histone modification and transcription regulation | [48] |
|  | *Zm00001d047993* | A0A1D6PFL0 | Putative inactive poly [ADP-ribose] polymerase SRO1 | 412 | Initiates DNA damage and other cellular responses | [49] |
|  | *Zm00001d033300* | A0A1D6KXK4 | Type IV inositol polyphosphate 5-phosphatase 11 | 347 | light-mediated growth processes or signaling pathways in addition to playing a major role in blue light signaling. | [50] |
|  | *Zm00001d008983* | A0A1D6FGZ8 | p-loop containing nucleoside triphosphate hydrolase superfamily protein | 563 | Signal transduction | [51] |
|  | *Zm00001d042114* | B6SGH6 | CYSTM domain-containing protein | 69 | Cell-cell communication and intracellular signalling | [52] |
|  | *Zm00001d025055* | K7U2B2 | Protein-serine/threonine phosphatase (EC 3.1.3.16) | 464 | Important components of stress signalling pathways | [53] |
|  | *Zm00001d028574* | A0A075T3H0 | Protein-serine/threonine phosphatase (EC 3.1.3.16) | 396 | Important components of stress signalling pathways | [53] |
|  | *Zm00001d033273* | A0A1D6KXB5 | Organic cation/carnitine transporter 7 | 273 | Regulation of pathways for nitrogen and/or carnitine supply during germination | [54] |
|  | *Zm00001d025964* | A0A1D6JBA8 | Putative homeobox DNA-binding and leucine zipper domain family protein | 268 | ABA and auxin signalling, embryogenesis and meristem function | [55] |
|  | *Zm00001d033718* | A0A1D6L1R0 | ACD11 homolog protein | 235 | Regulate membrane trafficking | [56] |
|  | *Zm00001d023664* | K7TFB6 | ABA-responsive protein | 272 | Involved in ABA signal transduction | [57] |
| **IV**  **CELLULAR PROCESS** | *Zm00001d012510* | A0A1D6G961 | Putative cytochrome P450 superfamily protein | 384 | Detoxification of xenobiotics, cellular metabolism and homeostasis | [58] |
|  | *Zm00001d008795* | A0A1D6FFL9 | UDP-glycosyltransferase 74B1 | 243 | Transfer of glycosyl molecule from activated nucleotide sugars and regulates their bioactivity, solubility and transport | [59] |
|  | *Zm00001d028999* | A0A060D2H9 | NAC domain containing protein 47 (NAC transcription factor) (Fragment) | 373 | Organ Size regulation | [60] |
|  | *Zm00001d028303* | A0A1D6JUA1 | Branched-chain-amino-acid aminotransferase (EC 2.6.1.42) | 374 | Play important role in leucine, isoleucine and valine metabolism | [61] |
|  | *Zm00001d017381* | B4FSX7 | (+)-neomenthol dehydrogenase (Carbonyl reductase 1) | 314 | Involved in monoterpenoid biosynthesis | [62] |
|  | *Zm00001d000438,Zm00001d021562* | B4F9D7 | Type III polyketide synthase B | 402 | Production of important secondary metabolites related to defense and pigmentation | [63] |
|  | *Zm00001d016255* | B4FXG7 | Heat stress transcription factor C-1 | 257 | Metabolism and redox homeostasis | [64] |
|  | *Zm00001d034978* | B6SXF1 | Farnesylated protein 2 | 151 | Cell cycle control | [65] |
|  | *Zm00001d019078* | A0A1D6HUY4 | AAA-ATPase ASD mitochondrial | 507 | Vesicle-mediated secretion, membrane fusion, cellular organelle biogenesis, and hypersensitive responses (HR) in plants | [66] |
|  | *Zm00001d009084* | C0HDZ4 | S-adenosyl-L-methionine-dependent methyltransferase superfamily protein | 262 | Involved in biosynthesis of nucleic acid, proteins, lipids, polysaccharides and secondary metabolites | [67] |
|  | *Zm00001d020702* | A0A1D6I5Q9 | Mitochondrial phosphate carrier protein 2 mitochondrial | 350 | Involved in disulphide bridge or protein–glutathione adduct reduction | [68] |
|  | *Zm00001d017240* | A0A1D6HD82 | Grx_C2.1-glutaredoxin subgroup I | 117 | Reduction of downstream pathways under oxidative stress | [69] |
|  | *Zm00001d028307* | A0A1D6JUA6 | Putative inactive poly [ADP-ribose] polymerase SRO1 | 431 | DNA repair, epigenetic regulation and transcription | [70] |
|  | *Zm00001d043741* | A0A1D6NEL8 | NPK1-related protein kinase-like protein | 475 | Cell plate formation | [71] |
| **V**  **HORMONES** | *Zm00001d047582* | Q7XYY1 | Hexosyltransferase (EC 2.4.1.-) | 345 | Phytohormone biosynthesis pathway | [72] |
|  | *Zm00001d003301* | A0A1D6E8G5 | C2 and GRAM domain-containing protein | 838 | ABA signalling pathway | [73] |
|  | *Zm00001d007341* | A0A1D6F5L3 | HVA22-like protein | 184 | ABA stress induced protein functions in vesicular trafficking and in the endoplasmic reticulum (ER) network in vivo | [74] |
|  | *Zm00001d038181* | B4FWH5 | Protein NRT1/ PTR FAMILY 3.1 | 602 | Transports plant hormones auxin (indole-3-acetic acid), abscisic acid (ABA), and gibberellin (GA), as well as secondary metabolites. | [75] |
|  | *Axel 2002* | A0A1D6HER1 | Homeobox-leucine zipper protein ATHB-6 | 235 | Regulator of the ABA signal pathway | [76] |
|  | *Zm00001d027443* | A0A1D6JM56 | Aldehyde oxygenase (deformylating) (EC 4.1.99.5) | 299 | Catalyzes the final steps of carotenoid catabolism and it is a key enzyme in the abscisic acid (ABA) biosynthesis | [77] |
|  | *Zm00001d010828* | A0A1D6FU87 | Alpha/beta-Hydrolases superfamily protein | 714 | Phytohormone and ligand receptors in the gibberellin, strigolactone, and karrikin signaling pathways in plants. | [78] |
|  | *Zm00001d012591* | A0A1D6G9Y2 | Putative nucleoredoxin 1 | 373 | Protects antioxidant enzymes such as catalase from ROS-induced oxidation | [79] |
|  | *Zm00001d037868* | A0A1D6M1D4 | Eukaryotic peptide chain release factor subunit 1-1 | 438 | Glucose and phytohormone (GA) response in plant growth and development | [80] |
|  | *Zm00001d033797* | A0A1D6L2B5 | Alpha/beta-Hydrolases superfamily protein | 105 | Phytohormone and ligand receptors in the gibberellin, strigolactone, and karrikin signaling pathways in plants. | [78] |
|  | *Zm00001d044529* | A0A1D6NMX1 | Protein NRT1/ PTR FAMILY 6.2 | 591 | Transporter of hormones like IAA, GA, JA and ABA | [81] |
|  | *Zm00001d037894* | A3KLI1 | Dehydrin DHN1 (RAB17 protein) | 168 | Regulation of ABA response | [82] |
| **UNKNOWN** | *Zm00001d013900* | A0A1D6GNF8 | Uncharacterized protein | 571 | Uncharacterised Protein | [83] |
|  | *Zm00001d019704* | A0A1D6HZV2 | Uncharacterized protein | 73 | Uncharacterised Protein | [84] |
|  | *Zm00001d046824* | K7VBL1 | Uncharacterized protein | 85 | Uncharacterised Protein | [85] |
|  | *Zm00001d044078* | B6SJA5 | Little protein 1 | 74 | Uncharacterised Protein | [86] |
|  | *Zm00001d013903* | A0A1D6GNG3 | Uncharacterized protein | 487 | Uncharacterised Protein | [87] |
|  | *Zm00001d031228* | A0A1D6KH07 | Uncharacterized protein | 164 | Uncharacterised Protein | [88] |
|  | *Zm00001d032138* | A0A1D6KNQ1 | Uncharacterized protein | 264 | Uncharacterised Protein | [89] |
|  | *Zm00001d050055* | K7U303 | Uncharacterized protein/putative glycine-rich cell wall structural protein 1 | 191 | Uncharacterised Protein | [90] |

**References**

[1] S. F. Hansen, J. Harholt, A. Oikawa, and H. v Scheller, “Plant Glycosyltransferases Beyond CAZy: A Perspective on DUF Families,” *Frontiers in Plant Science*, vol. 3, no. MAR, p. 59, 2012.

[2] I. Zelitch, N. P. Schultes, R. B. Peterson, P. Brown, and T. P. Brutnell, “High Glycolate Oxidase Activity Is Required for Survival of Maize in Normal Air,” *Plant Physiology*, vol. 149, no. 1, pp. 195–204, Jan. 2009.

[3] Y. Liu, Q. Song, D. Li, X. Yang, and D. Li, “Multifunctional roles of plant dehydrins in response to environmental stresses,” *Frontiers in Plant Science*, vol. 8, p. 1018, 2017.

[4] J. Medina, R. Catalá, and J. Salinas, “Developmental and Stress Regulation of RCI2A andRCI2B, Two Cold-Inducible Genes of Arabidopsis Encoding Highly Conserved Hydrophobic Proteins,” *Plant Physiology*, vol. 125, no. 4, pp. 1655–1666, Apr. 2001.

[5] M. Kumar, A. Brar, M. Yadav, A. Chawade, V. Vivekanand, and N. Pareek, “Chitinases—Potential Candidates for Enhanced Plant Resistance towards Fungal Pathogens,” *Agriculture*, vol. 8, no. 7, p. 88, 2018.

[6] K. Wei and X. Zhong, “Non-specific lipid transfer proteins in maize,” *BMC Plant Biology*, vol. 14, no. 1, pp. 1–18, 2014.

[7] M. W. Persans, J. Wang, and M. A. Schuler, “Characterization of Maize Cytochrome P450 Monooxygenases Induced in Response to Safeners and Bacterial Pathogens,” *Plant Physiology*, vol. 125, no. 2, pp. 1126–1138, 2001.

[8] X. Yuan, H. Wang, J. Cai, D. Li, and F. Song, “NAC transcription factors in plant immunity,” *Phytopathology Research 2019 1:1*, vol. 1, no. 1, pp. 1–13, 2019.

[9] N. Taki, Y Sasaki-Sekimoto, T. Obayashi et al., “12-Oxo-Phytodienoic Acid Triggers Expression of a Distinct Set of Genes and Plays a Role in Wound-Induced Gene Expression in Arabidopsis,” *Plant Physiology*, vol. 139, no. 3, pp. 1268–1283, 2005.

[10] H. W. Choi *et al.*, “A Role for a Menthone Reductase in Resistance against Microbial Pathogens in Plants,” *Plant Physiology*, vol. 148, no. 1, pp. 383–401, 2008.

[11] H. Maria and G. B. Fincher. "Structure-function relationships of β-D-glucan endo-and exohydrolases from higher plants." *Plant Molecular Biology*, vol. 47, no. 1, pp. 73-91, 2001.

[12] M. Naeem-ul-Hassan, Z. Zainal, C. J. Kiat, H. H. Monfared, and I. Ismail, “*Arabidopsis thaliana* SKP1 interacting protein 11 (At2g02870) negatively regulates the release of green leaf volatiles,” *RSC Advances*, vol. 7, no. 88, pp.55725-55733, 2017.

[13] “100278059 - Uncharacterized protein - Zea mays (Maize) - 100278059 gene & protein.” https://www.uniprot.org/uniprot/B6U3H7 (accessed Dec. 23, 2021).

[14] N. Liu, X. Zhang, Y. Sun et al., “Molecular evidence for the involvement of a polygalacturonase-inhibiting protein, GhPGIP1, in enhanced resistance to Verticillium and Fusarium wilts in cotton,” *Scientific Reports*, vol. 7, no. 1, pp. 1-18, 2017.

[15] J. Rösti, C.J. Barton, S. Albrecht et al., “UDP-Glucose 4-Epimerase Isoforms UGE2 and UGE4 Cooperate in Providing UDP-Galactose for Cell Wall Biosynthesis and Growth of Arabidopsis thaliana,” *The Plant Cell*, vol. 19, no. 5, pp. 1565–1579, 2007.

[16] M. Benedetti, I Verrascina, D Pontiggia et al., “Four Arabidopsis berberine bridge enzyme-like proteins are specific oxidases that inactivate the elicitor-active oligogalacturonides,” *The Plant Journal*, vol. 94, no. 2, pp. 260–273, 2018.

[17] “100274741 - Uncharacterized protein - Zea mays (Maize) - 100274741 gene & protein.” https://www.uniprot.org/uniprot/B6SI10 (accessed Dec. 23, 2021).

[18] A. Grover, “Plant Chitinases: Genetic Diversity and Physiological Roles,” *Critical reviews in Plant Sciences*, vol. 31, no. 1, pp. 57–73, 2012.

[19] P. Lemonnier, C. Galliard, F Veillet et al., “Expression of Arabidopsis sugar transport protein STP13 differentially affects glucose transport activity and basal resistance to Botrytis cinerea,” *Plant Molecular Biology*, vol. 85, no. 4, pp. 473–484, 2014.

[20] T. Gabaldón, D. Rainey, and M. A. Huynen, “Tracing the Evolution of a Large Protein Complex in the Eukaryotes, NADH:Ubiquinone Oxidoreductase (Complex I),” *Journal of Molecular Biology*, vol. 348, no. 4, pp. 857–870, 2005.

[21] X. Chen, A. Börner, X. Xin et al., “Comparative Proteomics at the Critical Node of Vigor Loss in Wheat Seeds Differing in Storability,” *Frontiers in Plant Science*, vol. 12, p. 1883, 2021.

[22] I. S. Day, V. S. Reddy, G. Shad Ali, and A. Reddy, “Analysis of EF-hand-containing proteins in Arabidopsis,” *Genome Biology 2002 3:10*, vol. 3, no. 10, pp. 1–24, 2002.

[23] S. Soyk, K. Šimková, E. Zürche et al., “The Enzyme-Like Domain of *Arabidopsis* Nuclear β-Amylases Is Critical for DNA Sequence Recognition and Transcriptional Activation,” *The Plant Cell*, vol. 26, no. 4, pp.1746-176, 2014.

[24] S. K. Cao, R. Liu, A. Sayye et al., “Regulator of Chromosome Condensation 1-Domain Protein DEK47 Functions on the Intron Splicing of Mitochondrial Nad2 and Seed Development in Maize,” *Frontiers in Plant Science*, vol. 12, p. 1612, 2021.

[25] M. A. Heim, “The Basic Helix-Loop-Helix Transcription Factor Family in Plants: A Genome-Wide Study of Protein Structure and Functional Diversity,” *Molecular Biology and Evolution*, vol. 20, no. 5, pp. 735-747, 2003.

[26] D. Shin, Y. D. Koo, J. Lee et al., “Athb-12, a homeobox-leucine zipper domain protein from Arabidopsis thaliana, increases salt tolerance in yeast by regulating sodium exclusion,” *Biochemical and Biophysical Research Communications*, vol. 323, no. 2, pp. 534–540, 2004.

[27] C. S. Barry, R. P. McQuinn, M.-Y. Chung, A. Besuden, and J. J. Giovannoni, “Amino Acid Substitutions in Homologs of the STAY-GREEN Protein Are Responsible for the *green-flesh* and *chlorophyll retainer* Mutations of Tomato and Pepper,” *Plant Physiology*, vol. 147, no. 1, pp. 179-187, 2008.

[28] W. Antosz, A Pfab, H. F. Ehrnsberger et al., “The Composition of the Arabidopsis RNA Polymerase II Transcript Elongation Complex Reveals the Interplay between Elongation and mRNA Processing Factors,” *The Plant Cell*, vol. 29, no. 4, pp. 854–870, 2017.

[29] E. R. Waters and E. Vierling, “Plant small heat shock proteins – evolutionary and functional diversity,” *New Phytologist*, vol. 227, no. 1, pp. 24–37, 2020.

[30] G. Janusz, A. Pawlik, U, Świderska-Burek et al., “Laccase Properties, Physiological Functions, and Evolution,” *International Journal of Molecular Sciences*, vol. 21, no. 3, p. 966, 2020.

[31] J. Kang, J. Park, H. Choi et al., “Plant ABC Transporters,” *Arabidopsis book/American Society of Plant Biologists*, vol. 2011, no. 9, p. e0153, 2011.

[32] I. Küfner and W. Koch, “Stress regulated members of the plant organic cation transporter family are localized to the vacuolar membrane,” *BMC Research Notes*, vol. 1, no. 1, pp. 1–10, 2008.

[33] S. Chowdhury, A. Basu, and S. Kundu, “Overexpression of a New Osmotin-Like Protein Gene (SindOLP) Confers Tolerance against Biotic and Abiotic Stresses in Sesame,” *Frontiers in Plant Science*, vol. 8, p.410, 2017.

[34] N. F. Cantelmo, R. G. von Pinho, and M. Balestre, “Genomic analysis of maize lines introduced in the early stages of a breeding programme,” *Plant Breeding*, vol. 136, no. 6, pp. 845–860, 2017.

[35] A. Chiappetta, A. Muto, L. Bruno, M. Woloszynska, M. van Lijsebettens, and M. B. Bitonti, “A dehydrin gene isolated from feral olive enhances drought tolerance in Arabidopsis transgenic plants,” *Frontiers in Plant Science*, vol. 6, p. 392, 2015.

[36] T. Zhou, C. Yue, J. Huang et al., “Genome-wide identification of the amino acid permease genes and molecular characterization of their transcriptional responses to various nutrient stresses in allotetraploid rapeseed,” *BMC Plant Biology*, vol. 20, no. 1, pp. 1–22, 2020.

[37] R. C. Mishra and A. Grover, “ClpB/Hsp100 proteins and heat stress tolerance in plants,” *Critical Reviews in Biotechnology*, vol. 36, no. 5, pp. 862–874, 2015.

[38] S. Ambawat, P. Sharma, N. R. Yadav, and R. C. Yadav, “MYB transcription factor genes as regulators for plant responses: an overview,” *Physiology and Molecular Biology of Plants*, vol. 19, no. 3, p. 307, 2013.

[39] B. Huang and J. Y. Liu, “A cotton dehydration responsive element binding protein functions as a transcriptional repressor of DRE-mediated gene expression,” *Biochemical and Biophysical Research Communications*, vol. 343, no. 4, pp. 1023–1031, 2006.

[40] O. Stein and D. Granot, “An overview of sucrose synthases in plants,” *Frontiers in Plant Science*, vol. 10, p. 95, 2019.

[41] P. Hatzopoulos, F. Fong, and Z. R. Sung, “Abscisic Acid Regulation of DC8, A Carrot Embryonic Gene,” *Plant Physiology*, vol. 94, no. 2, pp. 690–695, 1990.

[42] Z. Zhao and S. M. Assmann, “The glycolytic enzyme, phosphoglycerate mutase, has critical roles in stomatal movement, vegetative growth, and pollen production in Arabidopsis thaliana,” *Journal of Experimental Botany*, vol. 62, no. 14, pp. 5179–5189, 2011.

[43] F. Kaplan and C. L. Guy, “β-Amylase Induction and the Protective Role of Maltose during Temperature Shock,” *Plant Physiology*, vol. 135, no. 3, pp. 1674–1684, 2004.

[44] P. Aghaie and S. A. H. Tafreshi, “Central role of 70-kDa heat shock protein in adaptation of plants to drought stress,” *Cell Stress and Chaperones*, vol. 25, no. 6, pp. 1071–1081, 2020.

[45] F. Jia, B. Wu, H. Li, J. Huang, and C. Zheng, “Genome-wide identification and characterisation of F-box family in maize,” *Molecular Genetics and Genomics*, vol. 288, no. 11, pp. 559–577, 2013.

[46] Q. Huang, Y. Wang, B. Li et al., “TaNAC29, a NAC transcription factor from wheat, enhances salt and drought tolerance in transgenic Arabidopsis,” *BMC Plant Biology*, vol. 15, no. 1, pp. 1–15, 2015.

[47] R. Hu, J. Xiao, T. Gu et al., “Genome-wide identification and analysis of WD40 proteins in wheat (Triticum aestivum L.),” *BMC Genomics*, vol. 19, no. 1, pp. 1-13, 2018.

[48] L. Chen and H. Hellmann, “Plant E3 Ligases: Flexible Enzymes in a Sessile World,” *Molecular Plant*, vol. 6, no. 5, pp. 1388-1404, 2013.

[49] D. Rissel and E. Peiter, “Poly(ADP-Ribose) Polymerases in Plants and Their Human Counterparts: Parallels and Peculiarities,” *International Journal of Molecular Sciences 2019, Vol. 20, Page 1638*, vol. 20, no. 7, p. 1638, 2019.

[50] X. Chen, W. H. Lin, Y. Wang, S. Luan, and H. W. Xuea, “An Inositol Polyphosphate 5-Phosphatase Functions in PHOTOTROPIN1 Signaling in Arabidopis by Altering Cytosolic Ca2+,” *The Plant Cell*, vol. 20, no. 2, pp. 353–366, 2008.

[51] Y. Kawamura1, S. Ishii1, K. Asai1, A. Jp, N. Nagano3, and N. N. G. Jp, “Systematic Analyses of P-Loop Containing Nucleotide Triphosphate Hydrolase Superfamily Based on Sequence, Structure and Function,” *Genome Informatics*, vol. 14, pp. 581–582, 2003.

[52] Y. Xu *et al.*, “CYSTM, a Novel Non-Secreted Cysteine-Rich Peptide Family, Involved in Environmental Stresses in Arabidopsis thaliana,” *Plant and Cell Physiology*, vol. 59, no. 2, pp. 423–438, 2018.

[53] S. M. País, M. T. Téllez-Iñón, and D. A. Capiati, “Serine/Threonine Protein Phosphatases type 2A and their roles in stress signaling,” *Plant Signaling and Behaviour*, vol. 4, no. 11, pp. 1013–1015, 2009.

[54] C. Lelandais-Brière, M. Jovanovic, G. A. M. Torres et al., “Disruption of *AtOCT1*, an organic cation transporter gene, affects root development and carnitine-related responses in Arabidopsis,” *The Plant Journal*, vol. 51, no. 2, pp. 154-164, 2007.

[55] M. Elhiti and C. Stasolla, “Structure and function of homodomain-leucine zipper (HD-Zip) proteins,” *https://doi.org/10.4161/psb.4.2.7692*, vol. 4, no. 2, pp. 86–88, 2009.

[56] N. H. T. Petersen, J. Joensen, L. V. McKinney et al., “Identification of proteins interacting with Arabidopsis ACD11,” *Journal of Plant Physiology*, vol. 166, no. 6, pp. 661-666, 2009.

[57] L. Y. Liu, N. Li, C. P. Yao, S. S. Meng, and C. P. Song, “Functional analysis of the ABA-responsive protein family in ABA and stress signal transduction in Arabidopsis,” *Chinese Science Bulletin 2013 58:31*, vol. 58, no. 31, pp. 3721–3730, 2013.

[58] P. Manikandan and S. Nagini, “Cytochrome P450 Structure, Function and Clinical Significance: A Review,” *Current Drug Targets*, vol. 19, no. 1, Jan. 2018.

[59] J. Ross, Y. Li, E.-K. Lim, and D. J. Bowles, “Higher plant glycosyltransferases,” *Genome Biology 2001 2:2*, vol. 2, no. 2, pp. 1–6, 2001.

[60] Q. Chen, D. Jing, S. Wang et al., “The Putative Role of the NAC Transcription Factor EjNACL47 in Cell Enlargement of Loquat (Eriobotrya japonica Lindl.),” *Horticulturae 2021, Vol. 7, Page 323*, vol. 7, no. 9, p. 323, 2021.

[61] R. Diebold, J. Schuster, K. Däschner, and S. Binder, “The Branched-Chain Amino Acid Transaminase Gene Family in Arabidopsis Encodes Plastid and Mitochondrial Proteins,” *Plant Physiology*, vol. 129, no. 2, pp. 540–550, 2002.

[62] R. Kjonaas, C. Martinkus-Taylor, and R. Croteau3, “Metabolism of Monoterpenes: Conversion of l-Menthone to l-Menthol and d-Neomenthol by Stereospecific Dehydrogenases from Peppermint (Mentha piperita) Leaves,” *Plant Physiology*, vol. 69, no. 5, pp. 1013–1017, 1982.

[63] L. Meslet-Cladière,, L. Delage, C. J. J. Leroux et al., “Structure/Function Analysis of a Type III Polyketide Synthase in the Brown Alga Ectocarpus siliculosus Reveals a Biochemical Pathway in Phlorotannin Monomer Biosynthesis,” *The Plant Cell*, vol. 25, no. 8, pp. 3089–3103, 2013.

[64] H. chin Liu and Y. yung Charng, “Common and Distinct Functions of Arabidopsis Class A1 and A2 Heat Shock Factors in Diverse Abiotic Stress Responses and Development,” *Plant Physiology*, vol. 163, no. 1, pp. 276–290, 2013.

[65] D. Qian, D. Zhou, R. Ju, C. L. Cramer, and Z. Yang, “Protein farnesyltransferase in plants: molecular characterization and involvement in cell cycle control.,” *The Plant Cell*, vol. 8, no. 12, 1996.

[66] K. Baek, P. J. Seo, and C.-M. Park, “Activation of a mitochondrial ATPase gene induces abnormal seed development in Arabidopsis,” *Molecules and Cells 2011 31:4*, vol. 31, no. 4, pp. 361–369, 2011.

[67] J. G. Yu, G. H. Lee, and Y. D. Park, “Physiological role of endogenous S-adenosyl-L-methionine synthetase in Chinese cabbage,” *Horticulture, Environment, and Biotechnology 2012 53:3*, vol. 53, no. 3, pp. 247–255, 2012.

[68] N. Rouhier, J. Couturier and J. P. Jacquot, “Genome-wide analysis of plant glutaredoxin systems,” *Journal of Experimental Botany*, vol. 57, no. 8, pp. 1658-1696, 2006.

[69] C. Riondet, J. P. Desouris, J. G. Montoya, Y. Chartier, Y. Meyer, and J. P. Reichheld, “A dicotyledon-specific glutaredoxin GRXC1 family with dimer-dependent redox regulation is functionally redundant with GRXC2,” *Plant, Cell & Environment*, vol. 35, no. 2, pp. 360–373, 2012.

[70] R. S. Lamb, M. Citarelli, and S. Teotia, “Functions of the poly (ADP-ribose) polymerase superfamily in plants,” *Cellular and Molecular Life Sciences 2011 69:2*, vol. 69, no. 2, pp. 175–189, 2011.

[71] R. Nishihama, M. Ishikawa, S. Araki, T. Soyano, T. Asada, and Y. Machida, “The NPK1 mitogen-activated protein kinase kinase kinase is a regulator of cell-plate formation in plant cytokinesis,” *Genes & Development*, vol. 15, no. 3, pp. 352–363, 2001.

[72] X. Yue, X. G. Li, X. Q. Gao, X. Y. Zhao, Y. X. Dong, and C. Zhou, “The Arabidopsis phytohormone crosstalk network involves a consecutive metabolic route and circular control units of transcription factors that regulate enzyme-encoding genes,” *BMC Systems Biology*, vol. 10, no. 1, pp. 1–18, 2016.

[73] N. Mauri, M. Fernández-Marcos, C. Costas et al., “GEM, a member of the GRAM domain family of proteins, is part of the ABA signaling pathway,” *Scientific Reports 2016 6:1*, vol. 6, no. 1, pp. 1–11, 2016.

[74] W.-J. Guo and T.-H. David Ho, “An Abscisic Acid-Induced Protein, HVA22, Inhibits Gibberellin-Mediated Programmed Cell Death in Cereal Aleurone Cells” *Plant Physiology*, vol. 147, no. 4, pp. 1710-1722, 2008.

[75] Y. Chiba, T. Shimizu, S. Miyakawa et al., “Identification of Arabidopsis thaliana NRT1/PTR FAMILY (NPF) proteins capable of transporting plant hormones,” *Journal of Plant Research*, vol. 128, no. 4, pp. 679-686, 2015.

[76] A. Himmelbach, T. Hoffmann, M. Leube, B. Höhener, and E. Grill, “Homeodomain protein ATHB6 is a target of the protein phosphatase ABI1 and regulates hormone responses in Arabidopsis,” *The EMBO Journal*, vol. 21, no. 12, pp. 3029–3038, 2002.

[77] P. Colasuonno, I. Marcotuli, M. L. Lozito, R. Simeone, A. Blanco, and A. Gadaleta, “Characterization of Aldehyde Oxidase (AO) Genes Involved in the Accumulation of Carotenoid Pigments in Wheat Grain,” *Frontiers in Plant Science*, vol. 8, p. 863, 2017.

[78] J. T. Mindrebo, C. M. Nartey, Y. Seto, M. D. Burkart, and J. P. Noel, “Unveiling the functional diversity of the alpha/beta hydrolase superfamily in the plant kingdom,” *Current Opinion in Structural Biology*, vol. 41, pp. 233–246, 2016.

[79] S. Kneeshaw *et al.*, “Nucleoredoxin guards against oxidative stress by protecting antioxidant enzymes,” *Proceedings of the National Academy of Sciences*, vol. 114, no. 31, pp. 8414–8419, 2017.

[80] X. Zhou, P. Cooke, and L. Li, “Eukaryotic release factor 1-2 affects Arabidopsis responses to glucose and phytohormones during germination and early seedling development,” *Journal of Experimental Botany*, vol. 61, no. 2, pp. 357–367, 2010.

[81] C. Corratgé-Faillie and B. Lacombe, “Substrate (un)specificity of Arabidopsis NRT1/PTR FAMILY (NPF) proteins,” *Journal of Experimental Botany*, vol. 68, no. 12, pp. 3107–3113, 2017.

[82] A. Saleh, V. Lumbreras, C. Lopez, E. Dominguez-Puigjaner, D. Kizis, and M. Pagès, “Maize DBF1-interactor protein 1 containing an R3H domain is a potential regulator of DBF1 activity in stress responses,” *The Plant Journal*, vol. 46, no. 5, pp. 747–757, 2006.

[83] “103626123 - Uncharacterized protein - Zea mays (Maize) - 103626123 gene & protein.” https://www.uniprot.org/uniprot/A0A1D6GNF8 (accessed Dec. 23, 2021).

[84] “ZEAMMB73_Zm00001d019704 - Uncharacterized protein - Zea mays (Maize) - ZEAMMB73_Zm00001d019704 gene & protein.” https://www.uniprot.org/uniprot/A0A1D6HZV2 (accessed Dec. 23, 2021).

[85] “ZEAMMB73_Zm00001d046824 - Uncharacterized protein - Zea mays (Maize) - ZEAMMB73_Zm00001d046824 gene & protein.” https://www.uniprot.org/uniprot/K7VBL1 (accessed Dec. 23, 2021).

[86] “100279043 - Little protein 1 - Zea mays (Maize) - 100279043 gene & protein.” https://www.uniprot.org/uniprot/B6SJA5 (accessed Dec. 23, 2021).

[87] “ZEAMMB73_Zm00001d013903 - Uncharacterized protein - Zea mays (Maize) - ZEAMMB73_Zm00001d013903 gene & protein.” https://www.uniprot.org/uniprot/A0A1D6GNG3 (accessed Dec. 23, 2021).

[88] “103641047 - Uncharacterized protein - Zea mays (Maize) - 103641047 gene & protein.” https://www.uniprot.org/uniprot/A0A1D6KH07 (accessed Dec. 23, 2021).

[89] “103645275 - Uncharacterized protein - Zea mays (Maize) - 103645275 gene & protein.” https://www.uniprot.org/uniprot/A0A1D6KNQ1 (accessed Dec. 23, 2021).

[90] “103655312 - Uncharacterized protein - Zea mays (Maize) - 103655312 gene & protein.” https://www.uniprot.org/uniprot/K7U303 (accessed Dec. 23, 2021).
